# Supplementary material for: Seafaring and navigation in the Nordic Bronze Age: The application of an ocean voyage tool and boat performance data for comparing direct open water crossings with sheltered coastal routes
Source: PLoS One. 2025 Apr 2;20(4):e0320791. doi: 10.1371/journal.pone.0320791 (PMC11964266; doi:10.1371/journal.pone.0320791)
Supplement: Attachment — Submitted filename: PLOS_Fig.pdf [file pone.0320791.s002.pdf]

Click here to access/download;Figure;Fig1.tiff

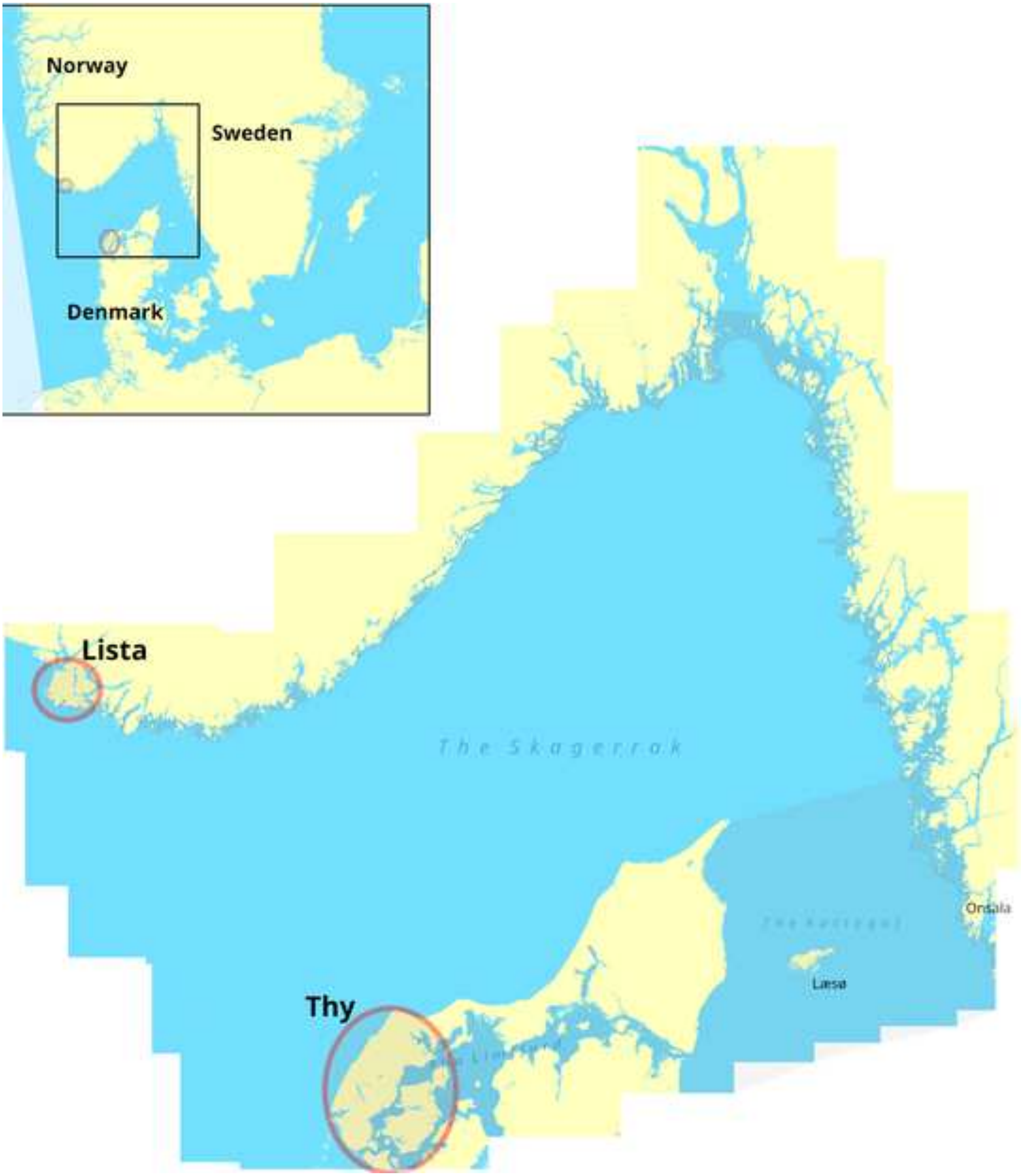

Click here to access/download;Figure;Fig2.tiff

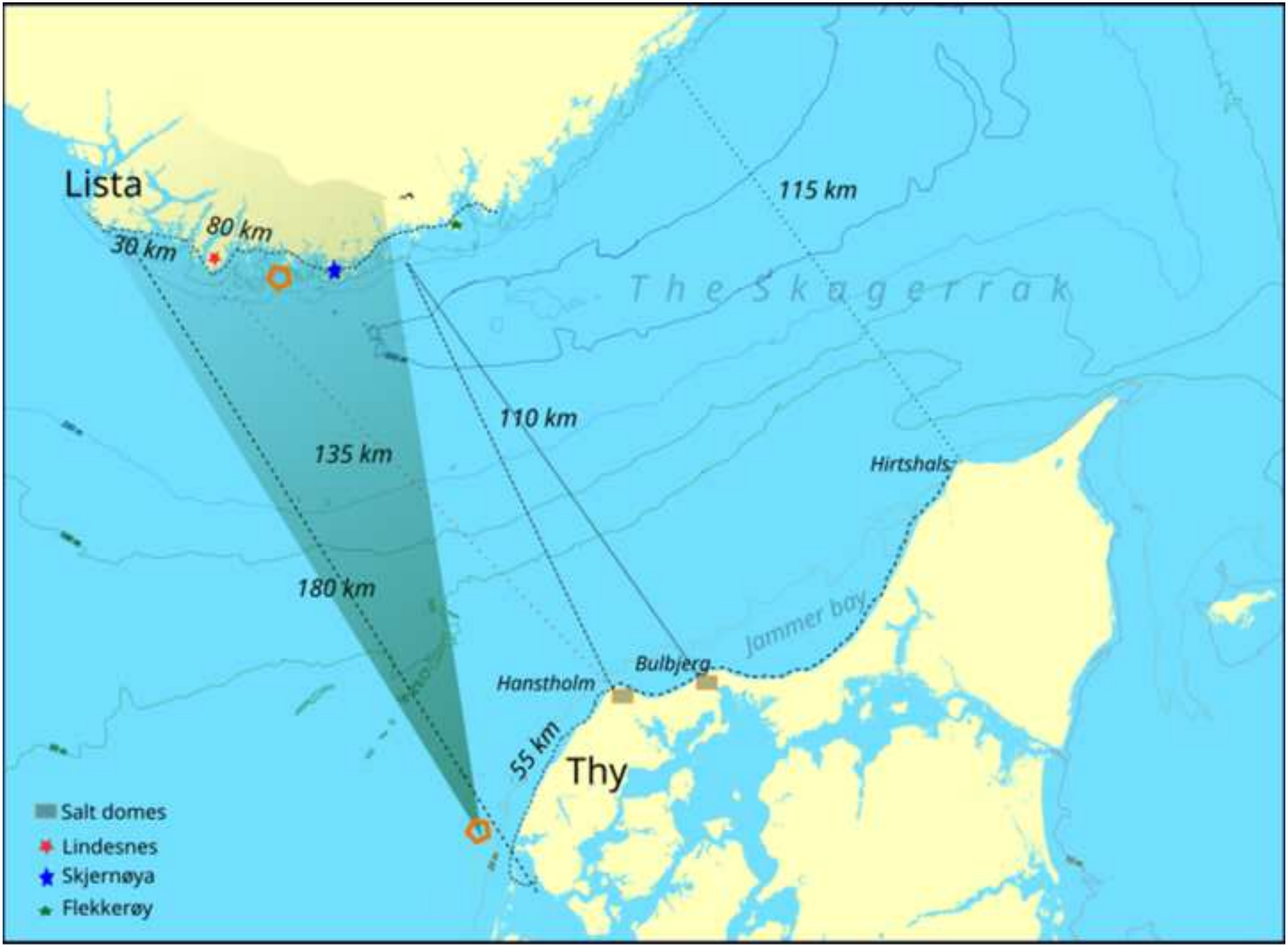

Click here to access/download;Figure;Fig3.tiff

View to the north from the hinterland of Hanstholm which reaches an elevation of c. 30 m in comparison to the former Littorina sea. Drawing, R. Christensen 1886 (after Bech 2018).

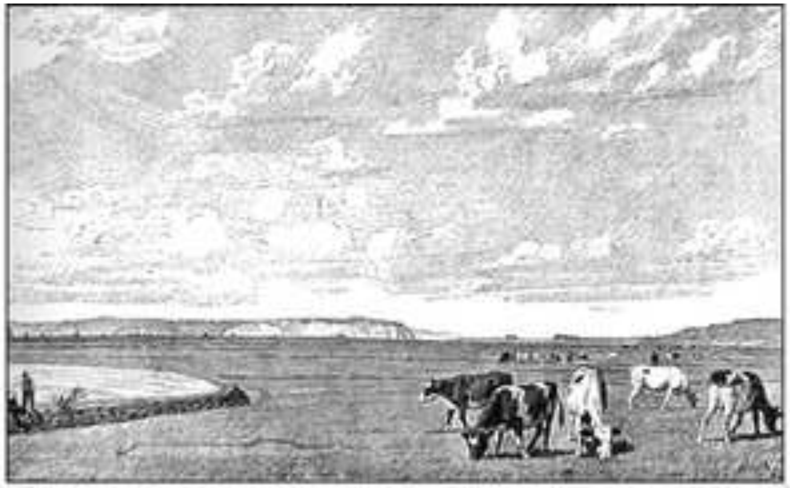

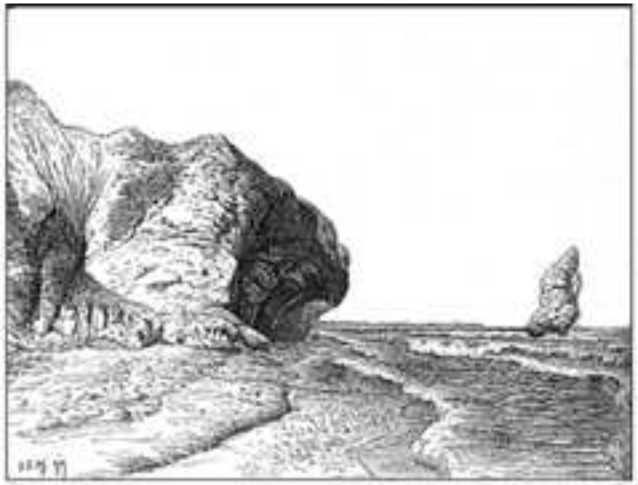

Bulbjerg 1899
A 47 m high rock providing a striking landmark. Birds nesting on the cliff might have aided seafarers to find land when visibility was poor (birdlandingplaces .eu).

Below.
Langed axe found during dredging in the northern part of Bjerre Enge. Votive deposit or dropped from a boat? Dated to c. 1950-1700 BC (Bech 2018; Vandkilde 1996).

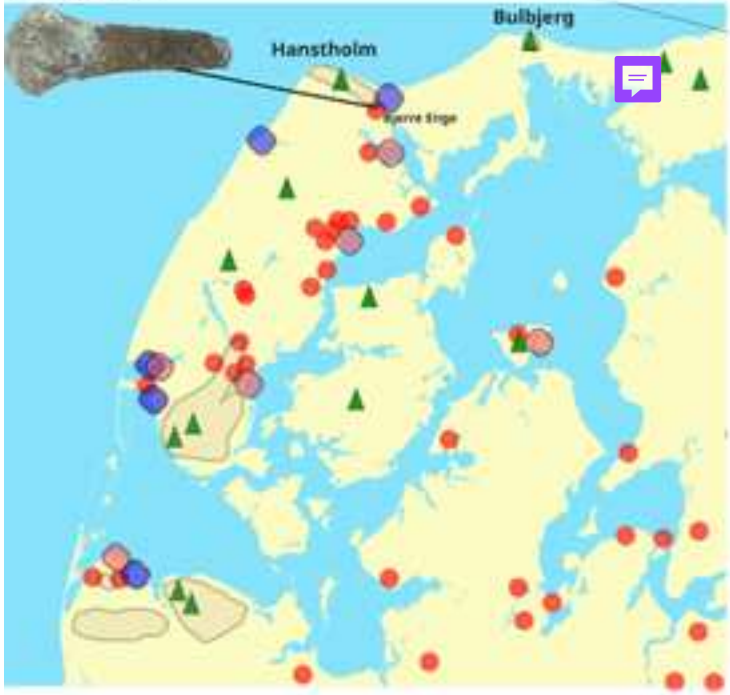

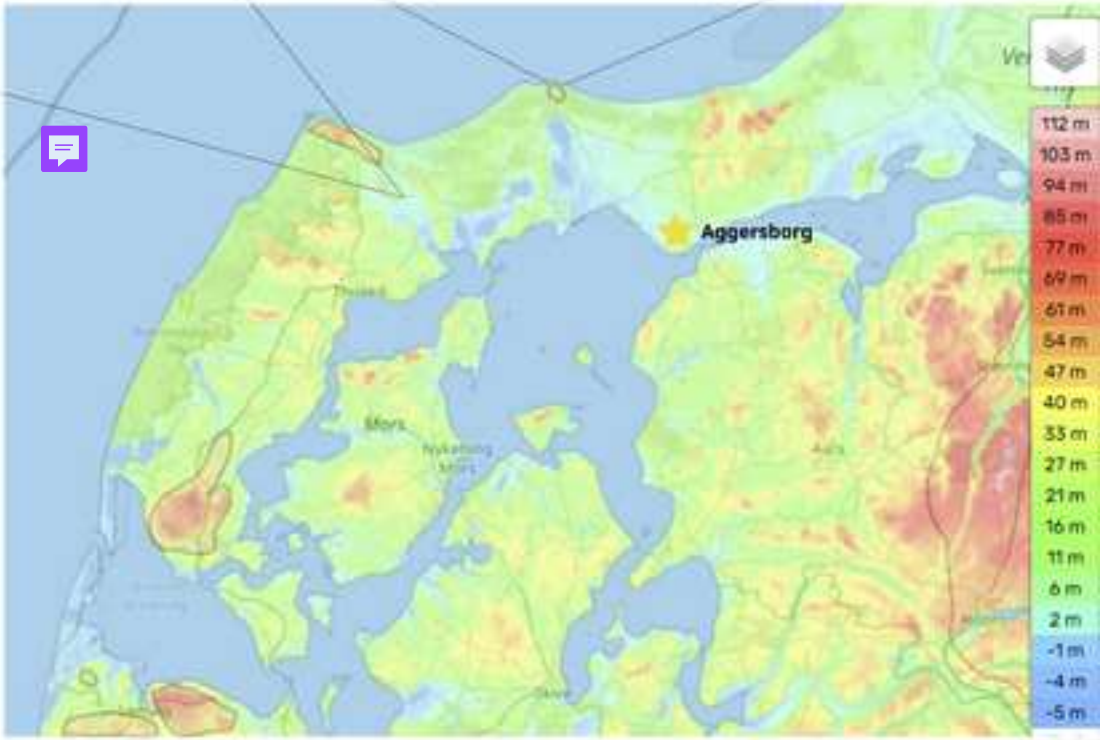

Modern topographic map, TessaDEM, ODbL (topographic-map.com)

- Location of 2-isled Early Bronze Age houses (after Beck & Rasmussen 2018).
- Potential landing sites, sheltered areas near houses.
- Area potentially providing shelter for boats attempting a crossing, c. 1500 BC
- Prominent features within the landscape providing navigational aid.
- Extent of prominent land features providing navigational aid.

Click here to access/download;Figure;Fig4.tiff

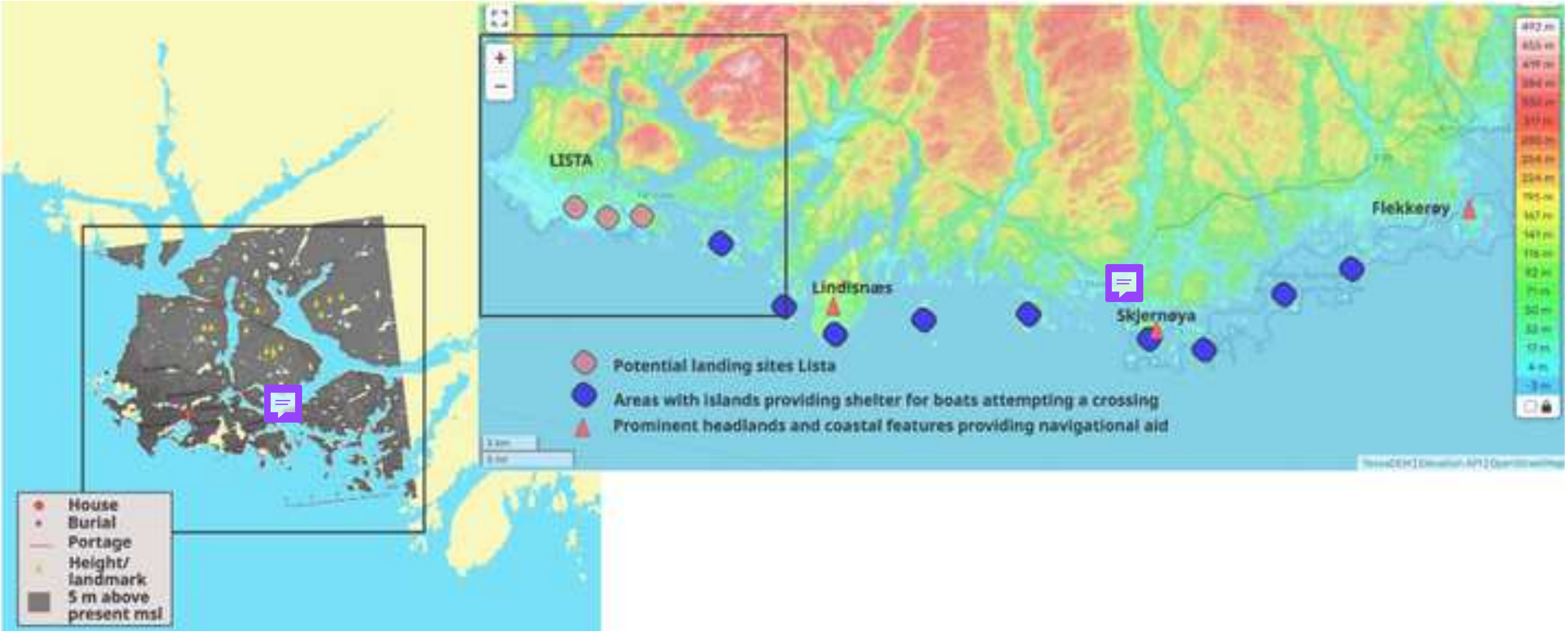

Click here to access/download;Figure;Fig5.tiff

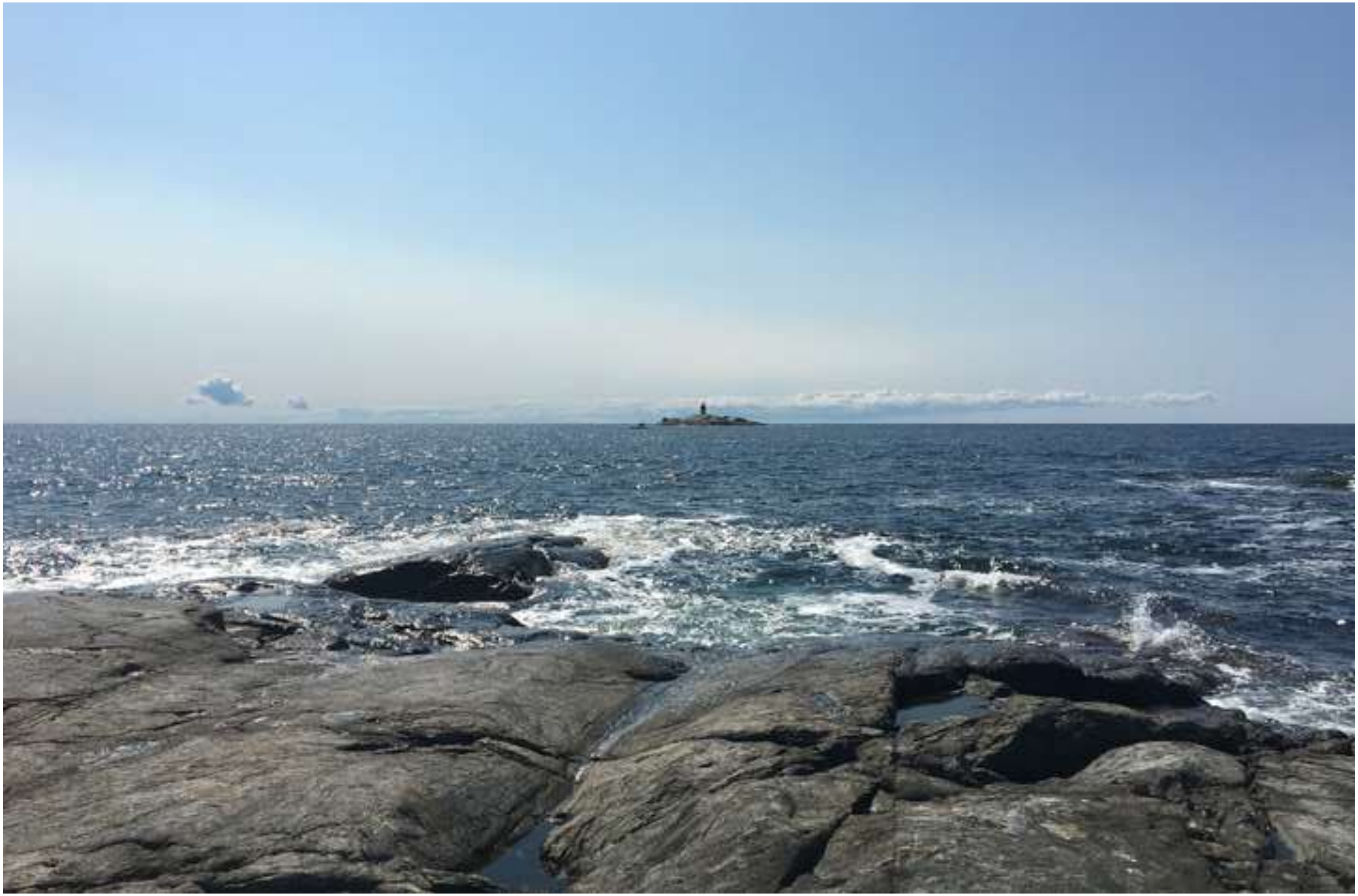

Click here to access/download;Figure;Fig6.tiff

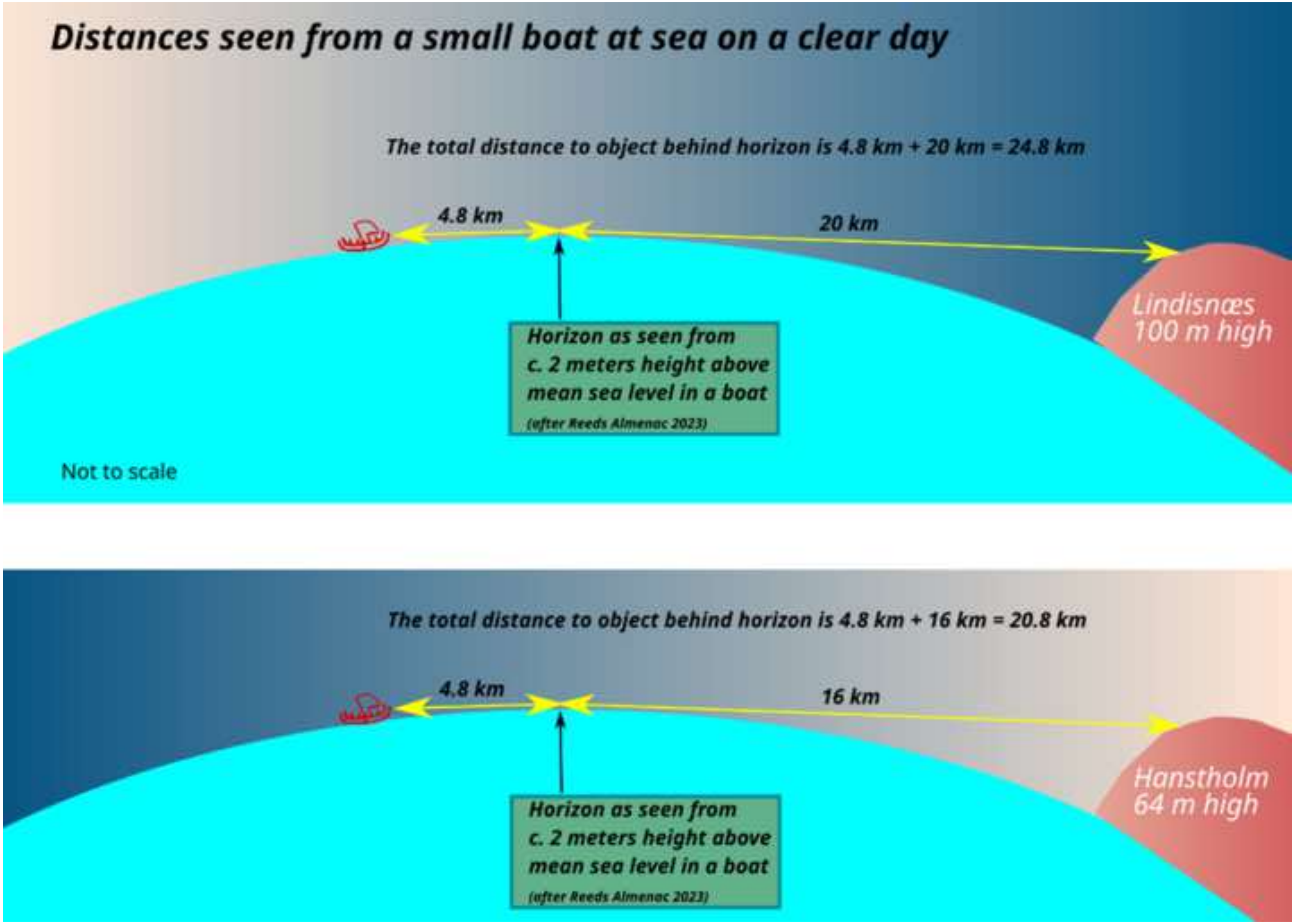

Click here to access/download;Figure;Fig7.tiff

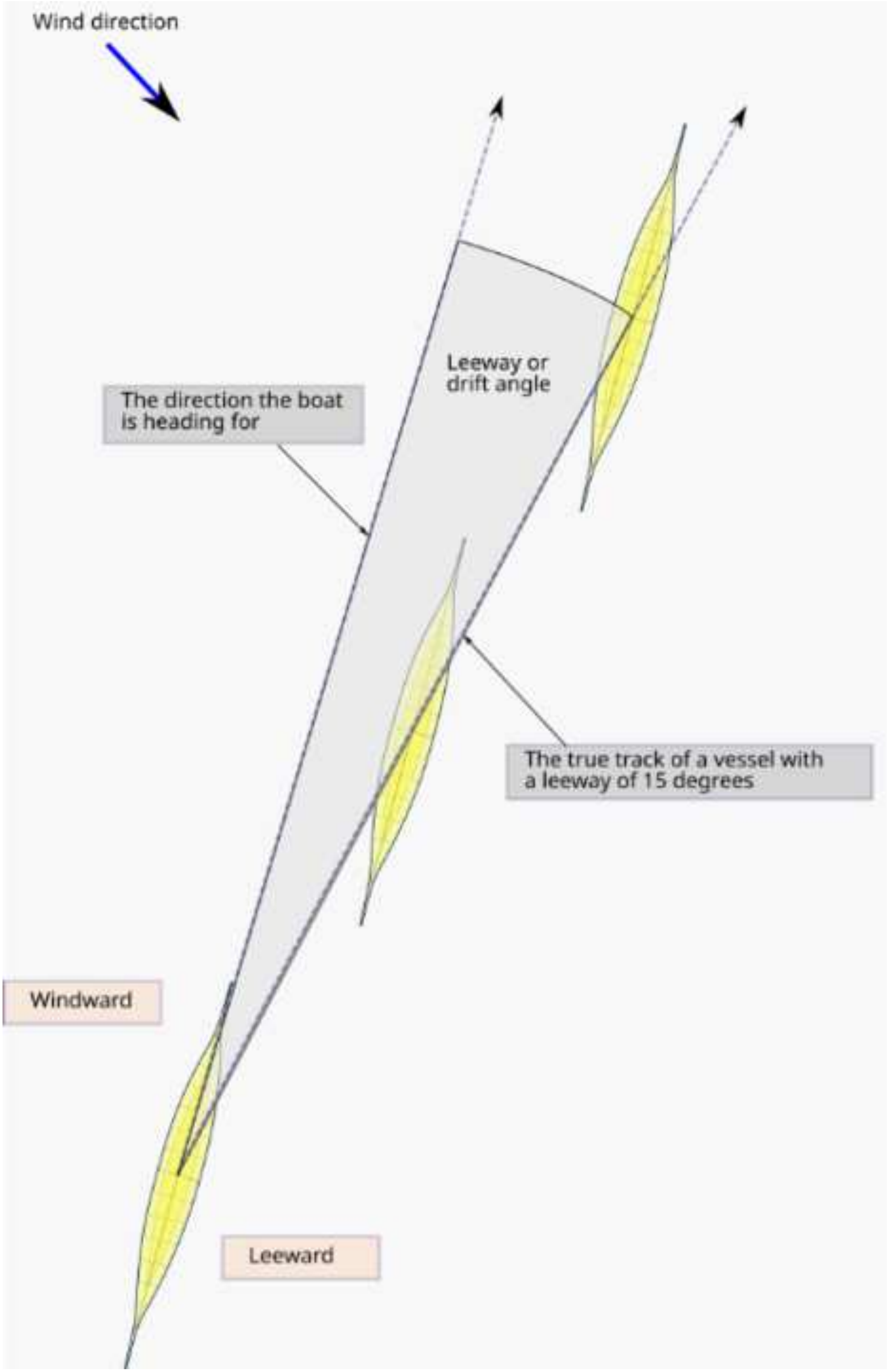

Click here to access/download;Figure;Fig8.tiff

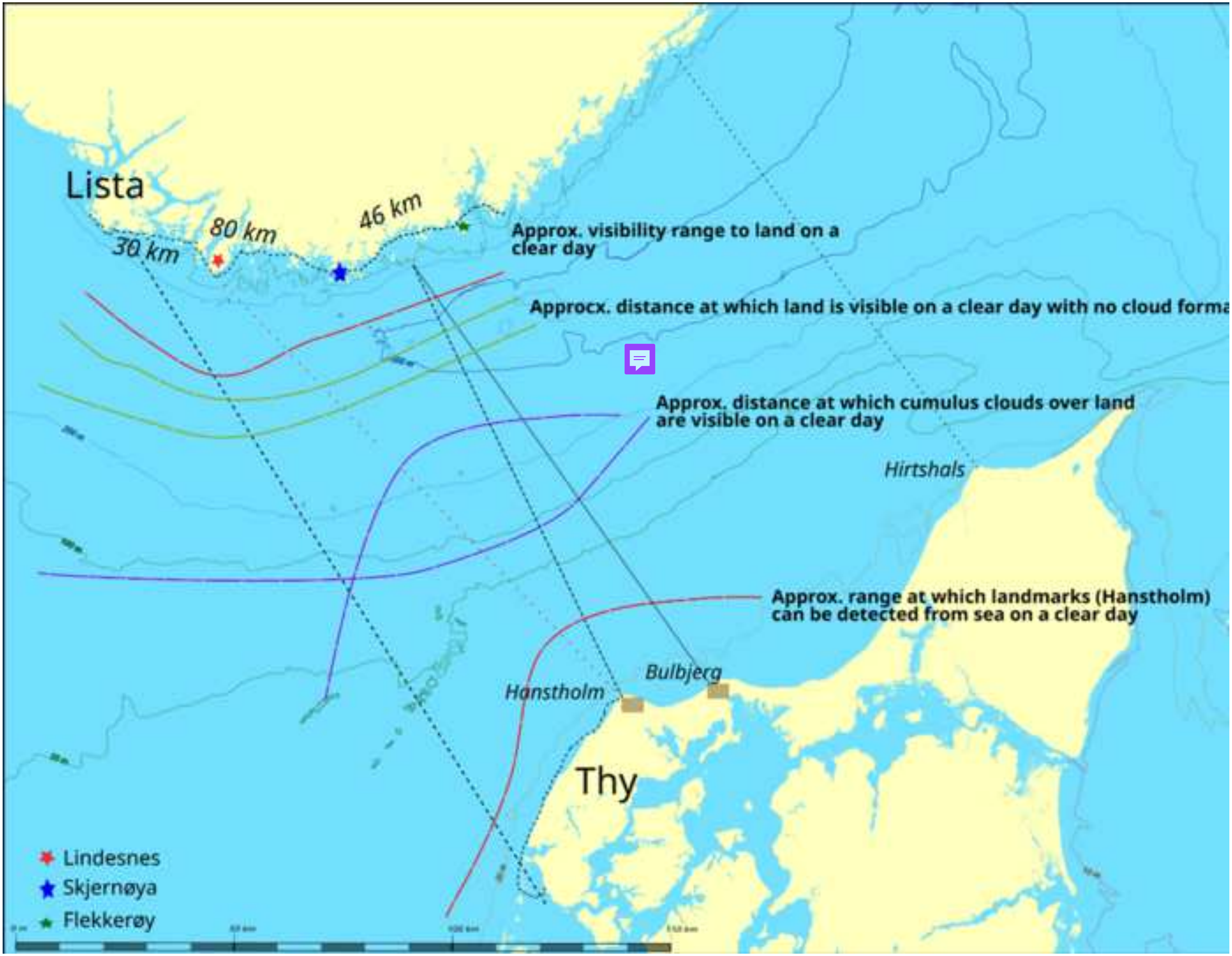

Click here to access/download;Figure;Fig9.tiff

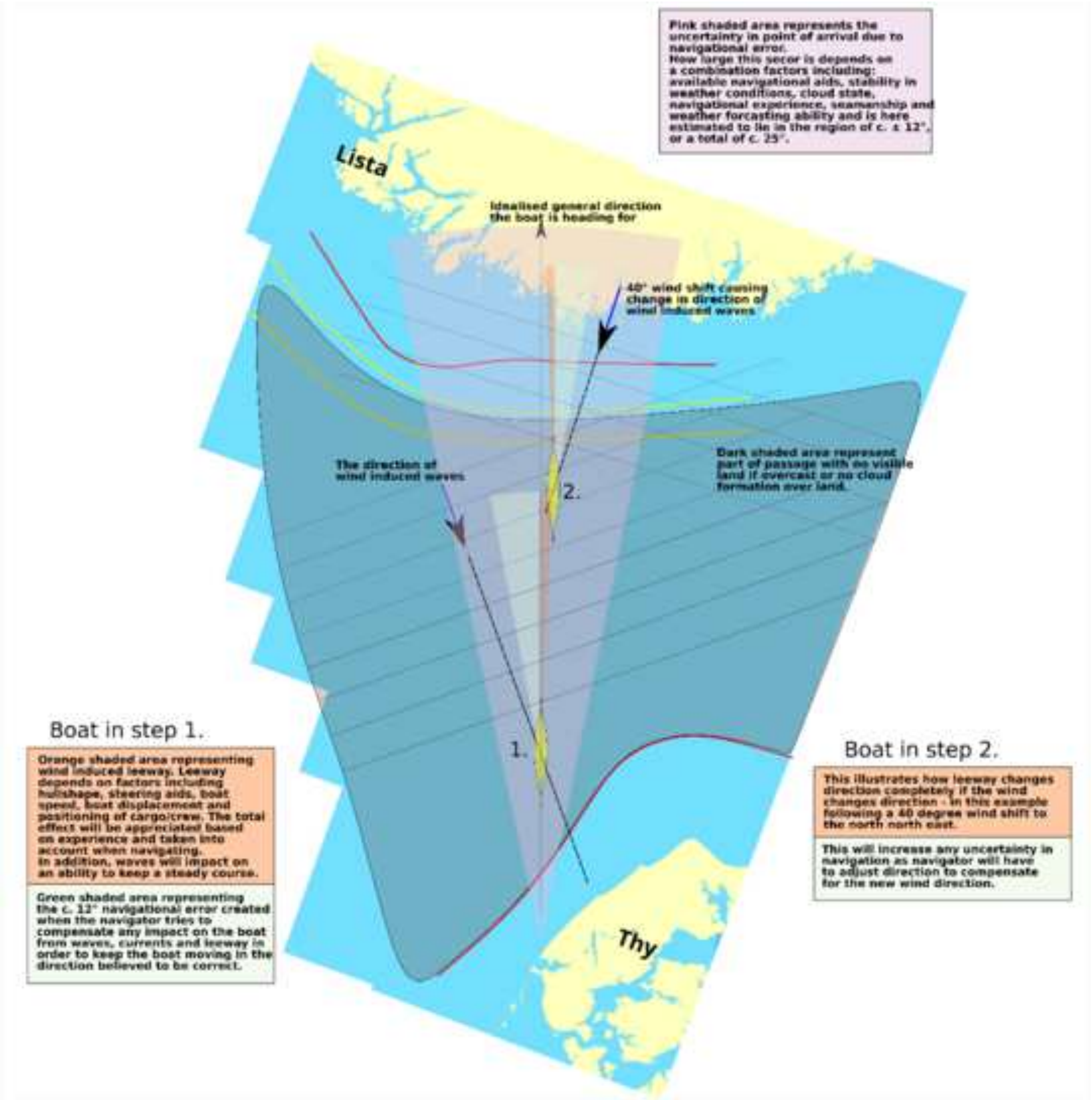

Click here to access/download;Figure;Fig10.tiff

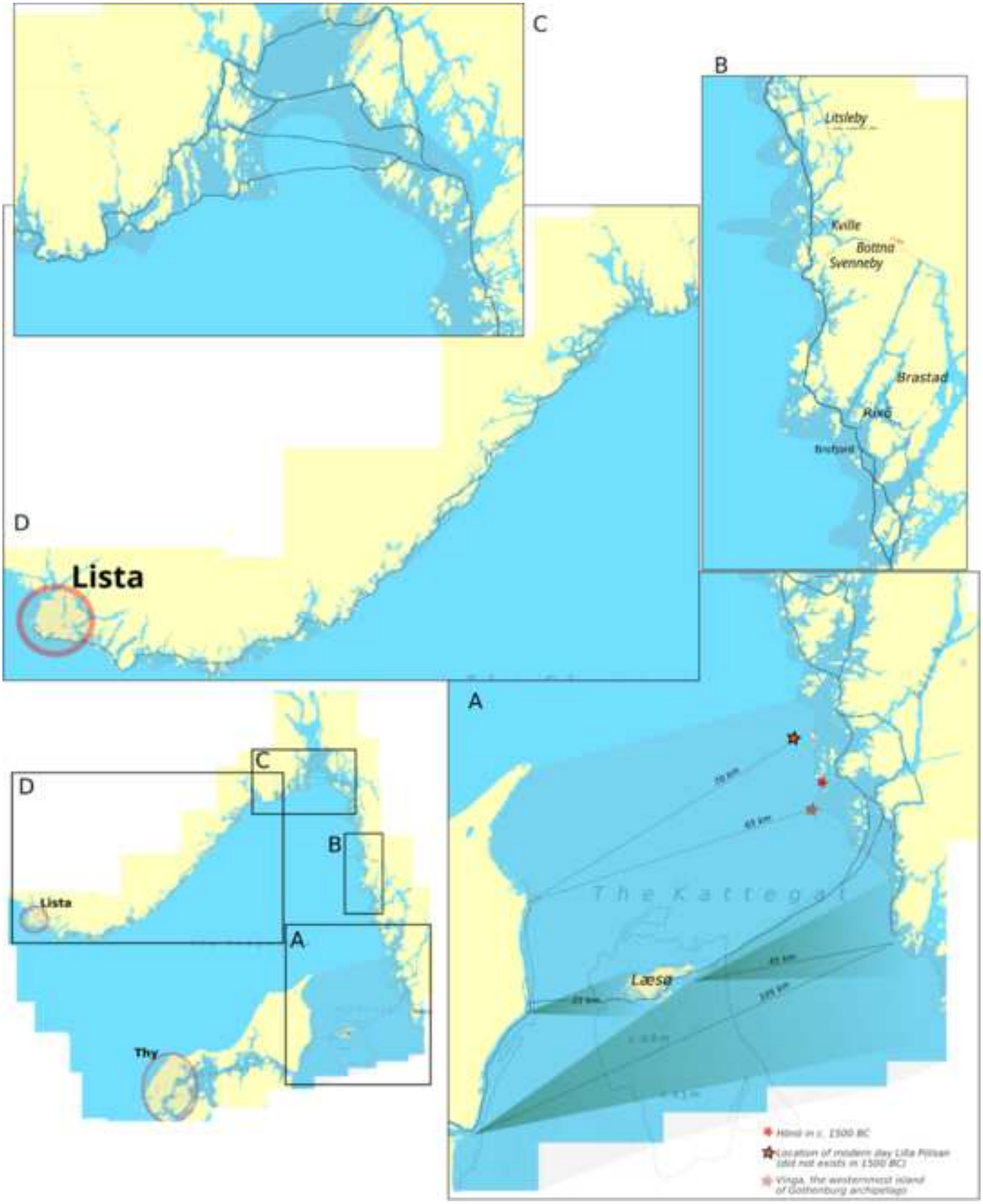

Click here to access/download;Figure;Fig11.tiff

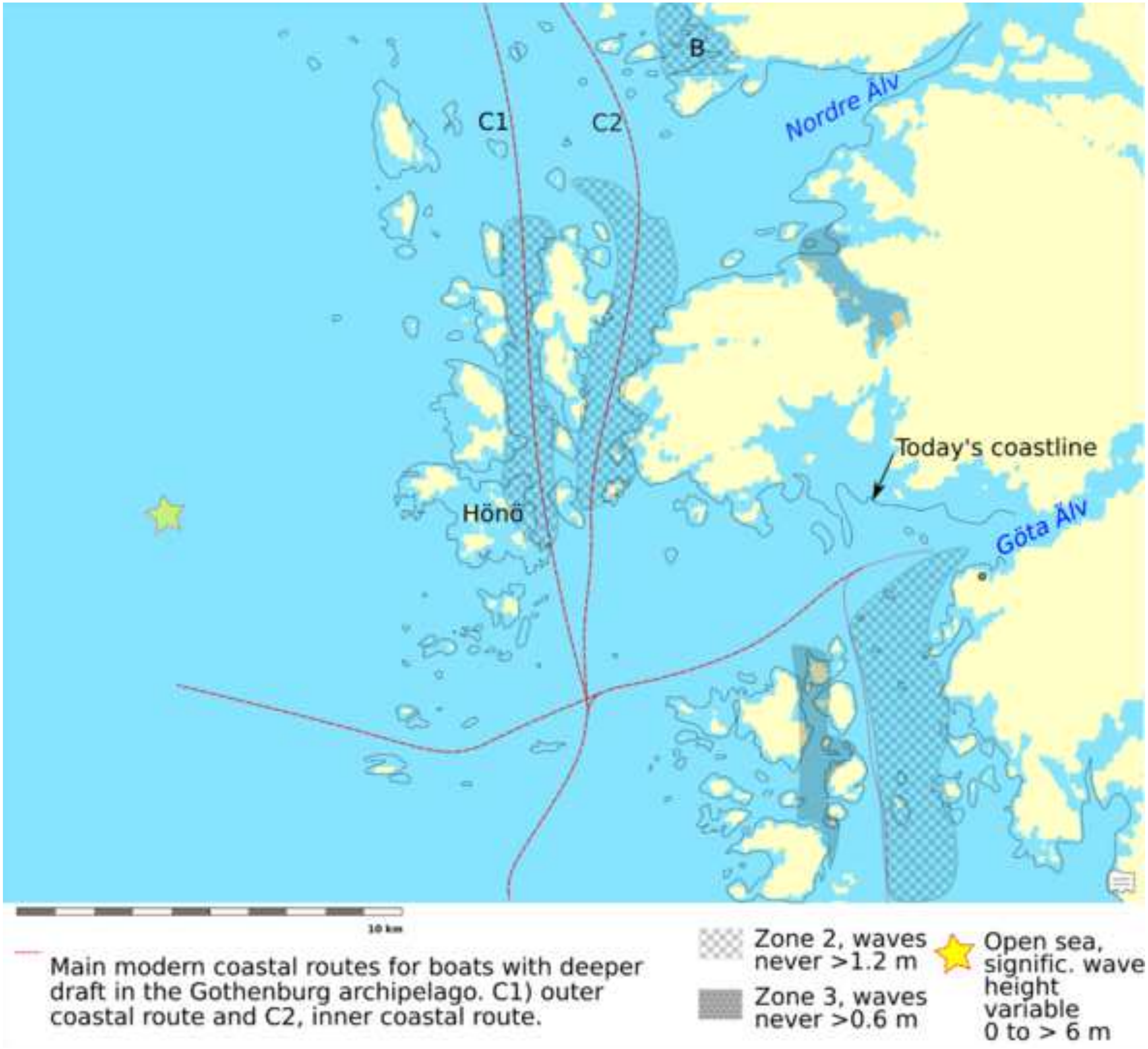

Click here to access/download;Figure;Fig12.tiff

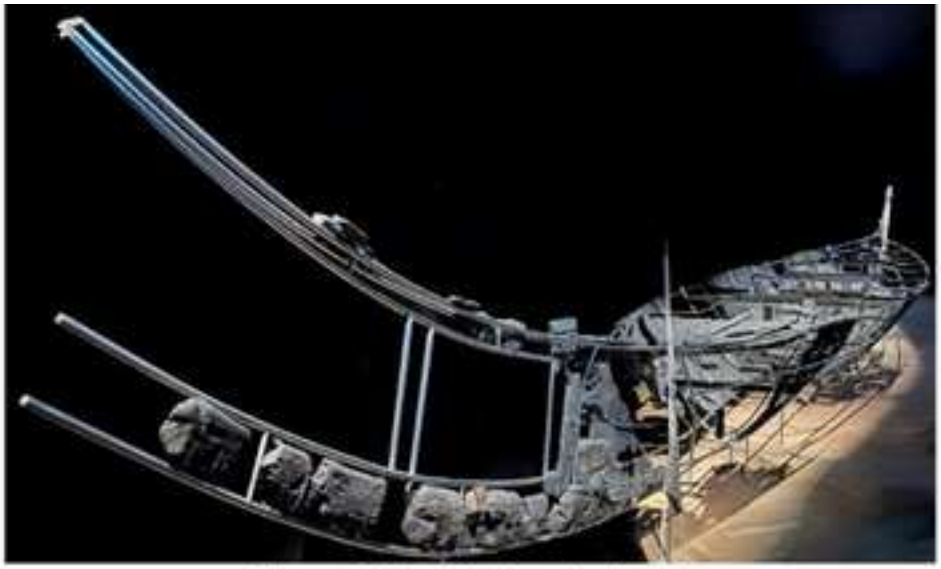

The c. 350 BC Hjortspring boat (Photo: Boel Bengtsson 2023

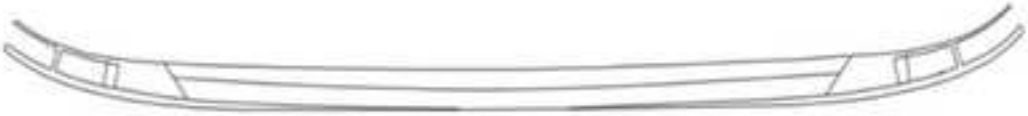

Line drawing of the Hjortspring boat (after Rosenberg 1937)

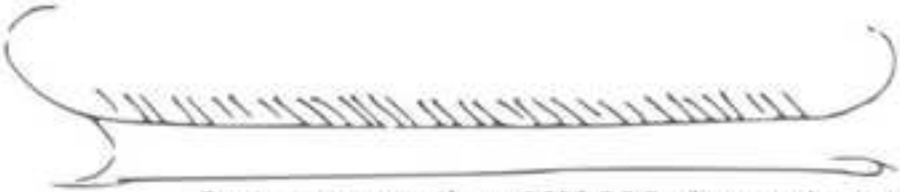

Boat engrave on the c. 1600 BC Rørby sword (after Kaul 1998)

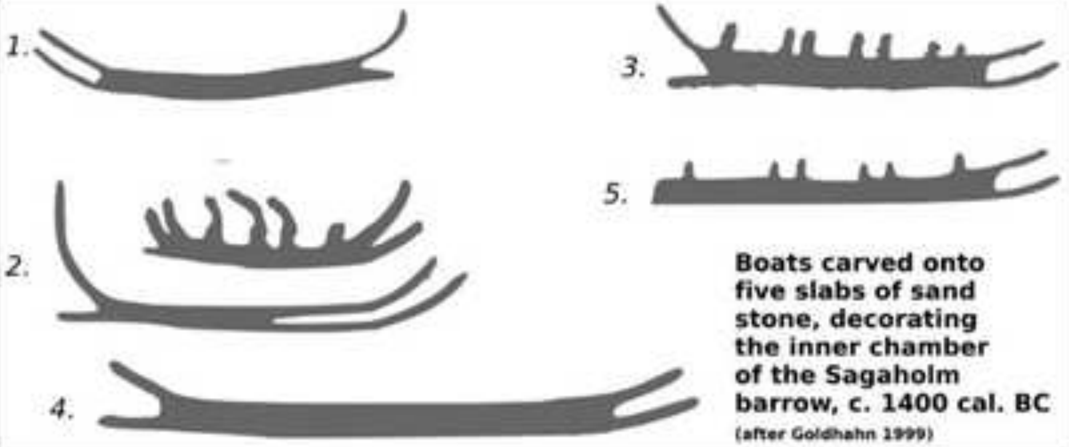

Boats carved onto five slabs of sand stone, decorating the inner chamber of the Sagaholm barrow, c. 1400 cal. BC (after Goldhahn 1999)

Rock carvings of boats belonging to a Scandinavian Bronze Age boatbuilding tradition (after Bengtsson 2017, Bengtsson et al. 2024)

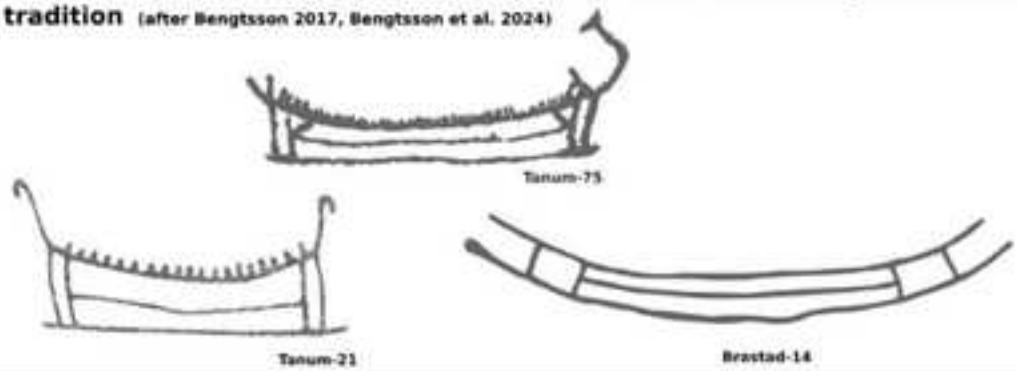

Click here to access/download;Figure;Fig13.tiff

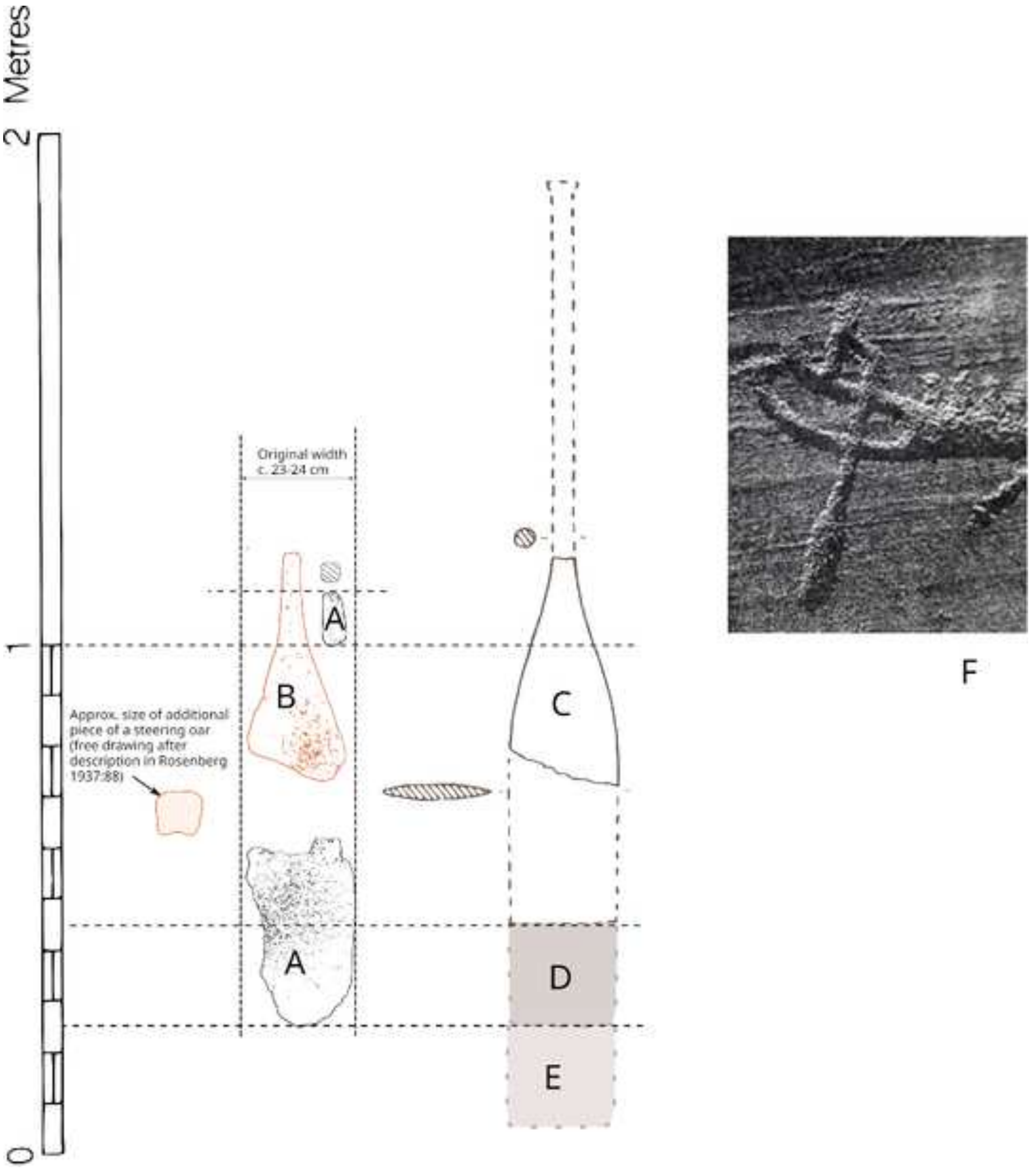

Click here to access/download;Figure;Fig14.tiff

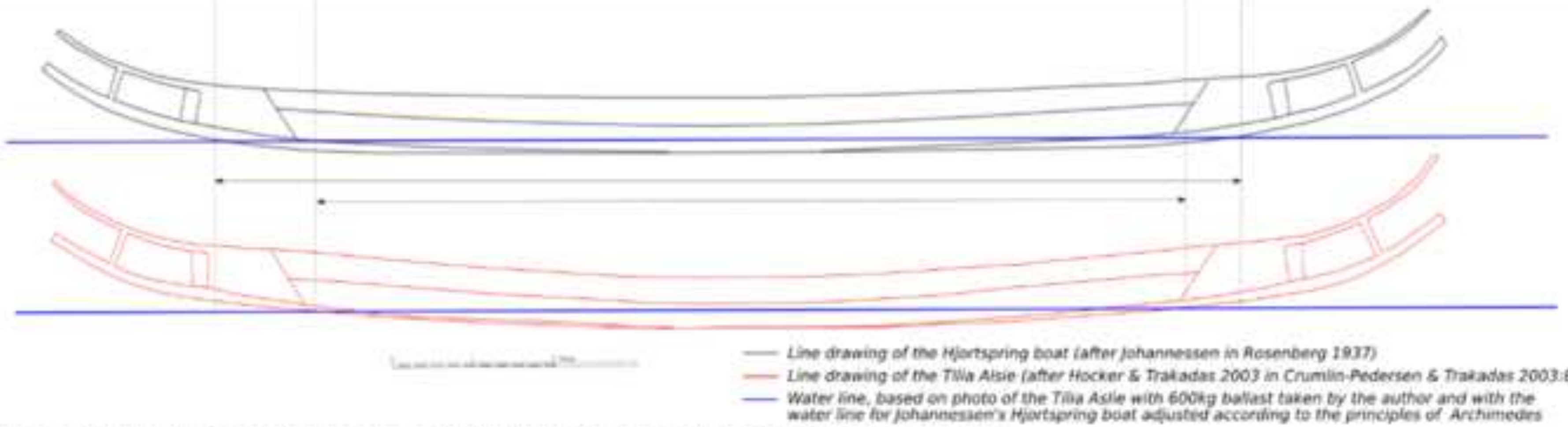

Difference in the hull shape between the original Hjortspring boat and the Tilia Alsie reconstruction:
Tilia Alsie: less course stability due to shorter water line. Needs cargo to compensate for this (create a longer water line). Hjortspring has a longer water line and more of the V-shaped endships aiding course stability.
Tilia Alsie: lower side but easier to turn

Click here to access/download;Figure;Fig15.tiff

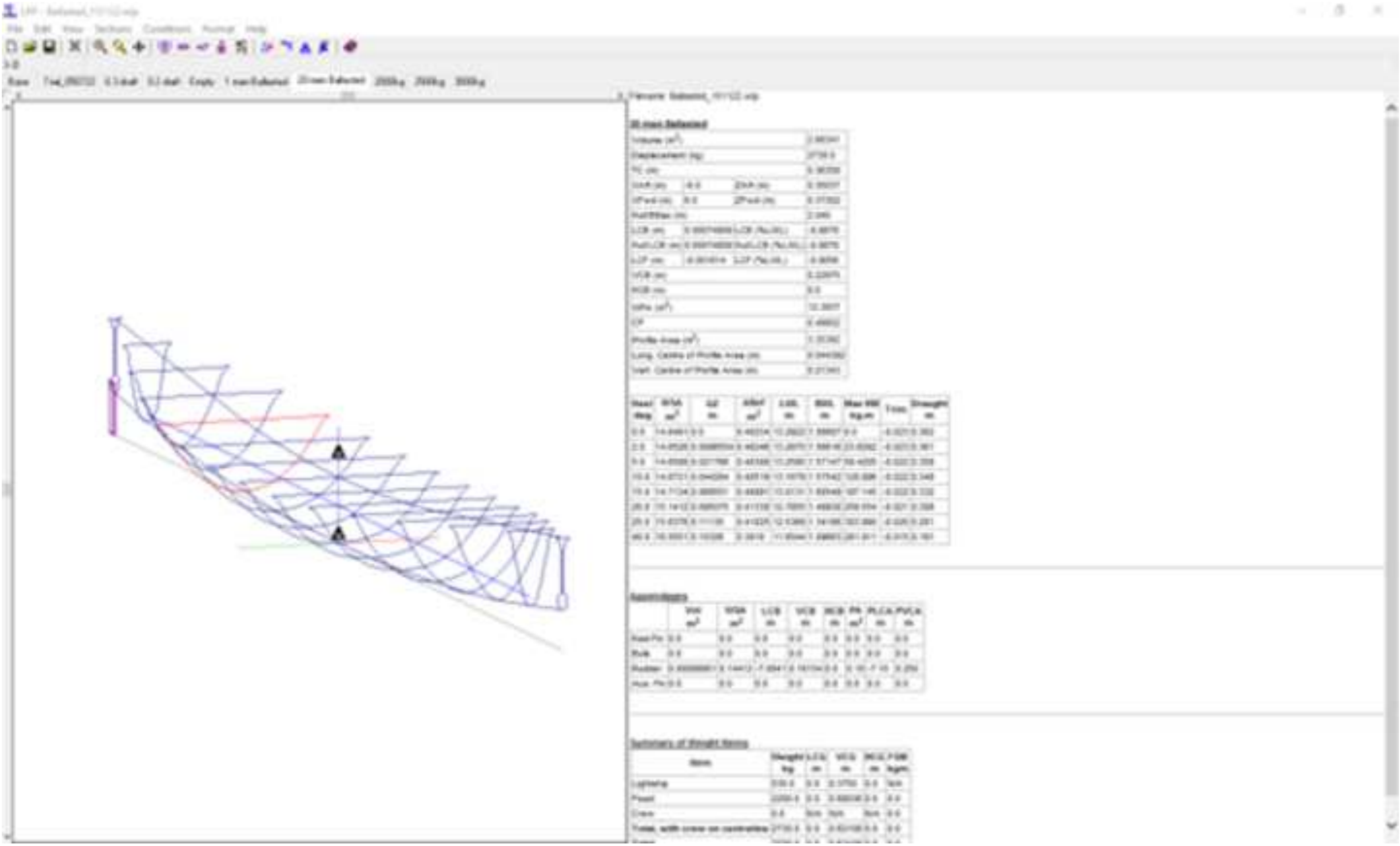

Click here to access/download;Figure;Fig16.tiff

No steering oars

Padding Boat Speed (knots)

True Wind Angle (degrees)	True Wind Speed (knots)						
	0	5	10	15	20	25	30
0	4.40	4.23	2.99	1.76	1.34	1.34	1.38
10	4.40	4.23	2.98	1.75	1.31	1.29	1.24
20	4.40	4.23	2.99	1.73	1.88	1.09	—
30	4.40	4.23	2.98	1.69	1.24	—	—
40	4.40	4.23	2.96	1.63	1.04	—	—
50	4.40	4.24	3.08	1.65	—	—	—
60	4.40	4.26	3.37	1.72	—	—	—
70	4.40	4.28	3.70	1.87	—	—	—
80	4.40	4.31	3.98	2.27	—	—	—
90	4.40	4.33	4.09	2.60	—	—	—
100	4.40	4.36	4.19	3.05	—	—	—
110	4.40	4.39	4.28	3.53	—	—	—
120	4.40	4.41	4.35	3.90	1.29	—	—
130	4.40	4.42	4.39	4.16	1.73	—	—
140	4.40	4.44	4.43	4.37	3.94	—	—
150	4.40	4.44	4.47	4.56	4.62	4.31	—
160	4.40	4.45	4.52	4.68	4.90	5.11	5.26
170	4.40	4.45	4.53	4.71	4.96	5.27	5.61
180	4.40	4.45	4.52	4.69	4.98	5.28	5.65

Leeway or Drift Angle (deg)

True Wind Angle (degrees)	True Wind Speed (knots)						
	0	5	10	15	20	25	30
0	0	0	0	0	0	0	0
10	0	0.49	1.97	4.61	7.39	10.59	14.24
20	0	0.93	3.29	7.78	11.83	19	-
30	0	1.27	4.28	10.32	17.41	-	-
40	0	1.53	5.07	12.47	22.27	-	-
50	0	1.7	5.43	13.99	-	-	-
60	0	1.8	5.34	14.79	-	-	-
70	0	1.83	5	14.78	-	-	-
80	0	1.79	4.56	13.18	-	-	-
90	0	1.7	4.31	11.63	-	-	-
100	0	1.55	3.98	9.43	-	-	-
110	0	1.38	3.59	7.78	-	-	-
120	0	1.12	3.14	6.3	24.36	-	-
130	0	0.87	2.65	5.2	18.65	-	-
140	0	0.61	2.15	4.2	7.2	-	-
150	0	0.37	1.62	3.21	4.92	7.35	-
160	0	0.18	1.09	2.27	3.4	4.5	5.65
170	0	0.06	0.55	1.3	1.99	2.67	3.26
180	0	0	0	0	0	0	0

One aft steering oar

Padding Boat Speed (knots)

True Wind Angle (degrees)	True Wind Speed (knots)						
	0	5	10	15	20	25	30
0	4.38	4.20	2.98	1.76	1.45	1.34	1.38
10	4.38	4.20	2.98	1.76	1.44	1.31	1.29
20	4.38	4.21	3.00	1.76	1.40	1.14	-
30	4.38	4.21	3.02	1.76	1.30	-	-
40	4.38	4.21	3.02	1.73	-	-	-
50	4.38	4.23	3.17	1.80	-	-	-
60	4.38	4.25	3.51	2.06	-	-	-
70	4.38	4.27	3.84	2.68	-	-	-
80	4.38	4.29	4.11	3.71	-	-	-
90	4.38	4.32	4.21	3.96	-	-	-
100	4.38	4.34	4.28	4.10	-	-	-
110	4.38	4.36	4.34	4.22	-	-	-
120	4.38	4.38	4.38	4.31	1.75	-	-
130	4.38	4.40	4.40	4.38	4.28	-	-
140	4.38	4.41	4.43	4.48	4.54	-	-
150	4.38	4.42	4.46	4.60	4.81	4.96	-
160	4.38	4.42	4.49	4.68	4.90	5.27	6.61
170	4.38	4.42	4.50	4.68	4.95	5.28	6.68
180	4.38	4.42	4.49	4.67	4.93	5.25	6.63

Leeway or Drift Angle (deg)

True Wind Angle (degrees)	True Wind Speed (knots)						
	0	5	10	15	20	25	30
0	0.00	0.00	0.00	0.00	0.00	0.00	0.00
10	0.00	0.17	0.83	2.46	5.30	8.55	12.41
20	0.00	0.32	1.40	4.57	9.20	17.48	-
30	0.00	0.46	1.76	7.26	12.02	-	-
40	0.00	0.57	2.04	9.84	-	-	-
50	0.00	0.65	2.19	10.95	-	-	-
60	0.00	0.69	2.11	10.58	-	-	-
70	0.00	0.71	1.92	7.51	-	-	-
80	0.00	0.69	1.76	3.94	-	-	-
90	0.00	0.65	1.68	3.21	-	-	-
100	0.00	0.58	1.57	2.88	-	-	-
110	0.00	0.49	1.43	2.57	-	-	-
120	0.00	0.40	1.26	2.26	18.98	-	-
130	0.00	0.30	1.07	1.94	3.19	-	-
140	0.00	0.21	0.85	1.63	2.48	-	-
150	0.00	0.12	0.61	1.28	1.84	2.42	-
160	0.00	0.06	0.39	0.90	1.35	1.70	2.19
170	0.00	0.02	0.19	0.47	0.77	1.06	1.29
180	0.00	0.00	0.00	0.00	0.00	0.00	0.00

Two steering oars, one at each end

Padding Boat Speed (knots)

True Wind Angle (degrees)	True Wind Speed (knots)						
	0	5	10	15	20	25	30
0	4.32	4.15	2.98	1.75	1.34	1.34	1.38
10	4.32	4.15	2.98	1.75	1.32	1.31	1.31
20	4.32	4.15	2.98	1.76	1.71	1.18	-
30	4.32	4.15	3.00	1.76	1.81	-	-
40	4.32	4.16	3.01	1.76	1.19	-	-
50	4.32	4.17	3.15	1.88	-	-	-
60	4.32	4.19	3.47	2.21	-	-	-
70	4.32	4.21	3.80	2.77	-	-	-
80	4.32	4.23	4.06	3.65	-	-	-
90	4.32	4.26	4.15	3.90	-	-	-
100	4.32	4.28	4.22	4.05	-	-	-
110	4.32	4.31	4.28	4.17	1.90	-	-
120	4.32	4.33	4.32	4.25	4.09	-	-
130	4.32	4.34	4.34	4.33	4.23	-	-
140	4.32	4.35	4.36	4.42	4.50	4.52	-
150	4.32	4.36	4.40	4.54	4.75	5.00	5.24
160	4.32	4.36	4.43	4.62	4.89	5.21	5.58
170	4.32	4.36	4.44	4.62	4.89	5.22	5.60
180	4.32	4.36	4.43	4.61	4.87	5.19	5.56

Leeway or Drift Angle (deg)

True Wind Angle (degrees)	True Wind Speed (knots)						
	0	5	10	15	20	25	30
0	0.00	0.00	0.00	0.00	0.00	0.00	0.00
10	0.00	0.10	0.54	1.86	3.97	6.88	10.30
20	0.00	0.20	0.97	3.29	6.19	15.79	-
30	0.00	0.28	1.28	4.79	9.02	-	-
40	0.00	0.35	1.48	6.32	18.79	-	-
50	0.00	0.40	1.56	7.40	-	-	-
60	0.00	0.43	1.50	6.78	-	-	-
70	0.00	0.44	1.39	4.34	-	-	-
80	0.00	0.43	1.28	2.44	-	-	-
90	0.00	0.40	1.21	2.13	-	-	-
100	0.00	0.36	1.12	1.93	-	-	-
110	0.00	0.30	1.00	1.77	16.65	-	-
120	0.00	0.24	0.86	1.60	2.51	-	-
130	0.00	0.18	0.70	1.41	2.13	-	-
140	0.00	0.13	0.54	1.16	1.71	2.37	-
150	0.00	0.08	0.39	0.88	1.33	1.67	2.02
160	0.00	0.04	0.24	0.58	0.92	1.23	1.46
170	0.00	0.01	0.12	0.29	0.49	0.69	0.87
180	0.00	0.00	0.00	0.00	0.00	0.00	0.00

Leeway above 1 degree

Paddling starting to become difficult

Paddling not possible

Drift starting to be a concern

Large drift, boat difficult or impossible to handle

Click here to access/download;Figure;Fig17.tiff

The Tilia Alsie viewed from the side, based on photograph.
Illustrates the relatively high positioning of the crew.

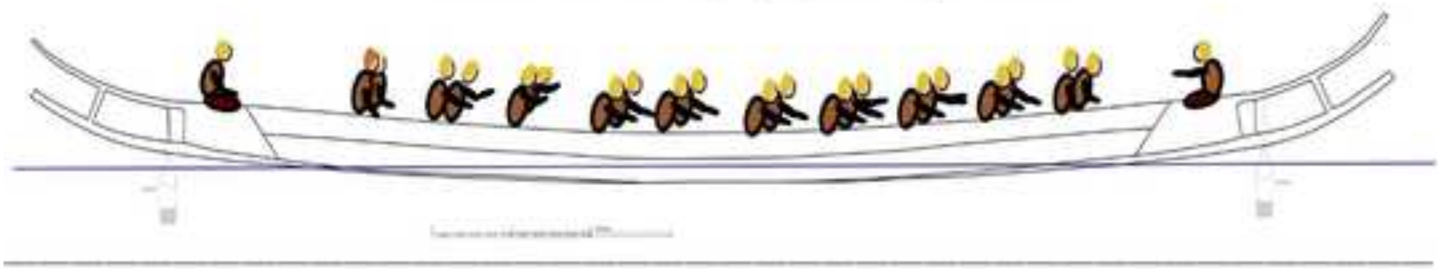

Propulsion using paddles: paddling force not constant
since a crew will get tired. Sail generates constant power

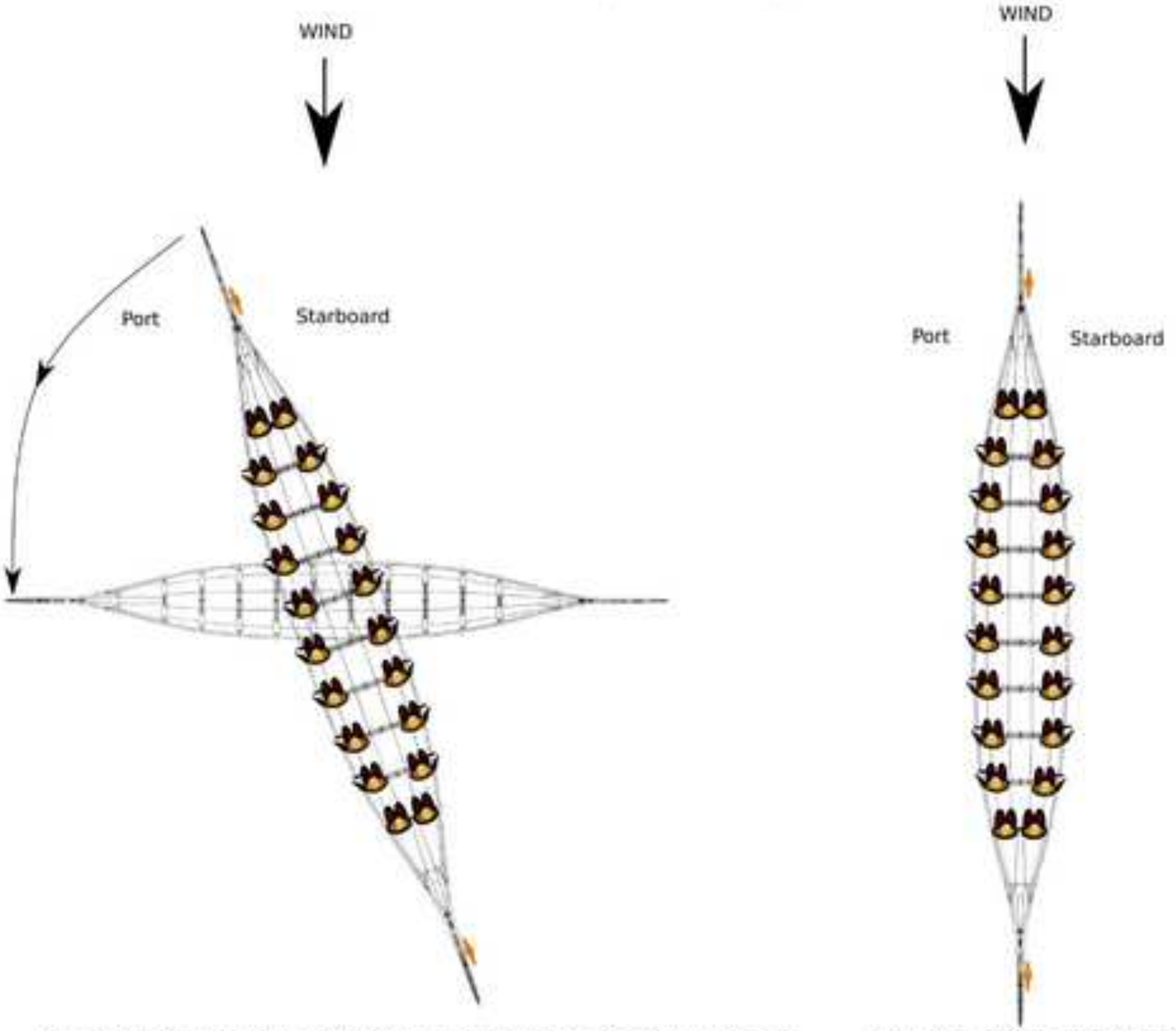

With the wind pressing on the starboard side of the boat, the crew on this side will have to stop or reduce their paddling speed in order for the boat to maintain an even direction. The crew on the port side will have to paddle with more force if the boat is to maintain speed and course or heading. If not, the boat will veer to the left.

When the crew is unable to hold the boat against the wind (due to fatigue), it will broach, i.e. it will veer further to the left until it lies sideways to the wind. This will expose its side, the lowest point of the vesse,l to the wind threatening the boat to be swamped with water from waves hitting it from the side.

For this reason the two steering oars are of paramount importance in ensuring the power generated by the paddlers on each side remains more even and can be used to propell the boat forwards (meaning starboard side crew might need to paddle only slightly less and makes it less heavy for the port crew.

Crew on starboard and port side paddling at even speed and with even force between the two sides. The boat goes forwards and direction of movemen[illegible] maintained, aided by the two steering oar[illegible].

Click here to access/download;Figure;Fig18.tiff

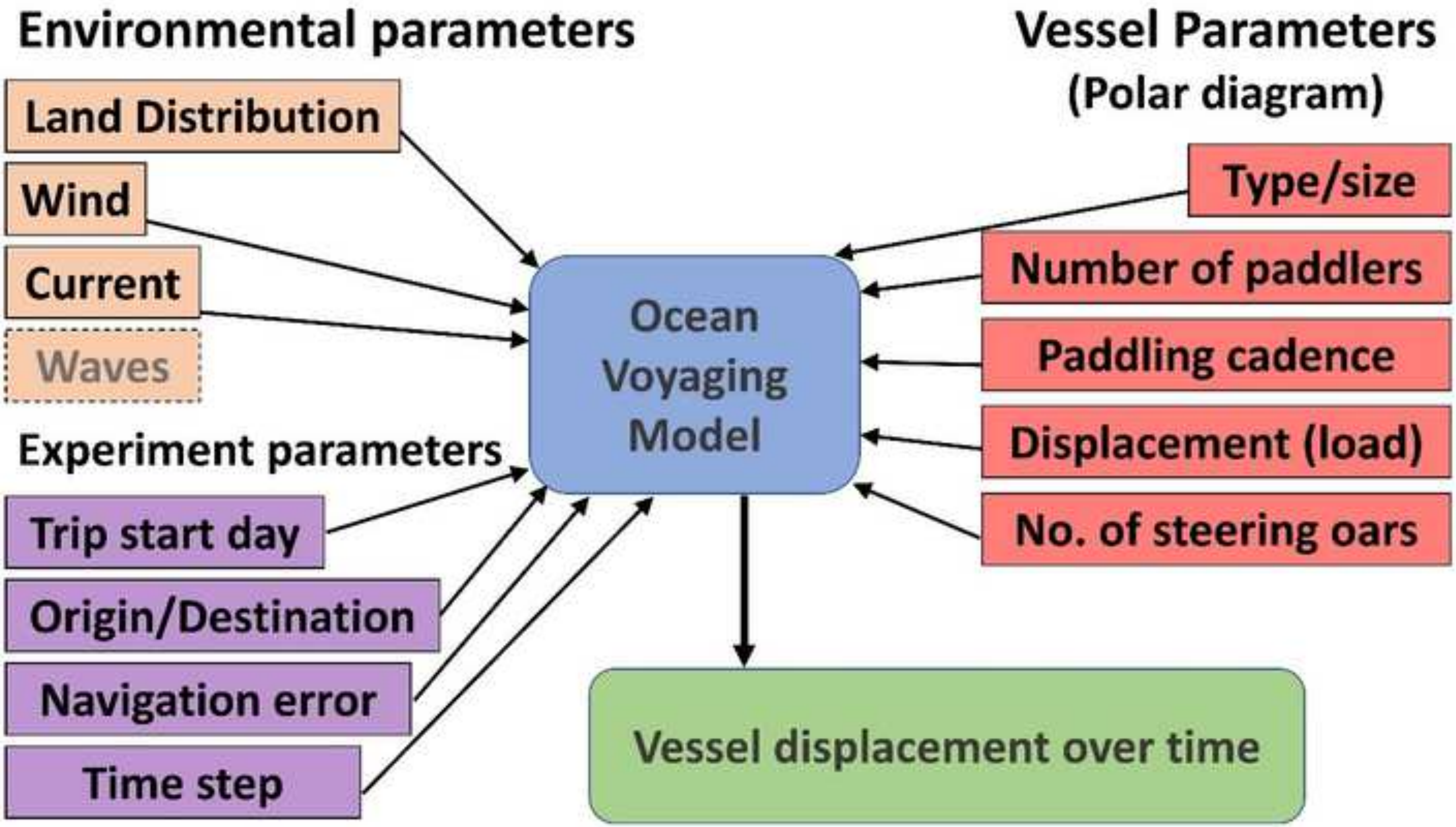

Click here to access/download;Figure;Fig19.tiff

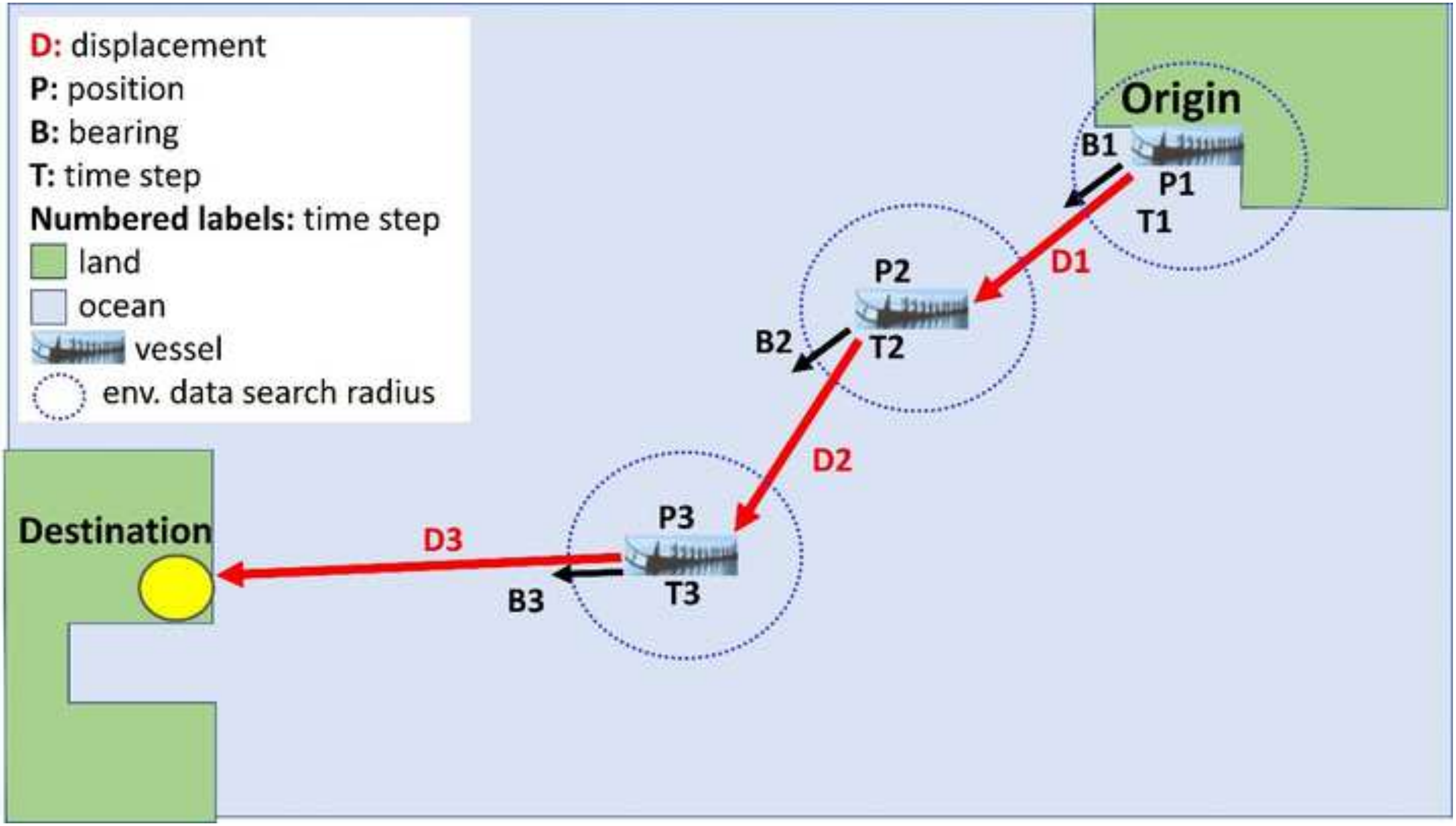

Click here to access/download;Figure;Fig20.tiff

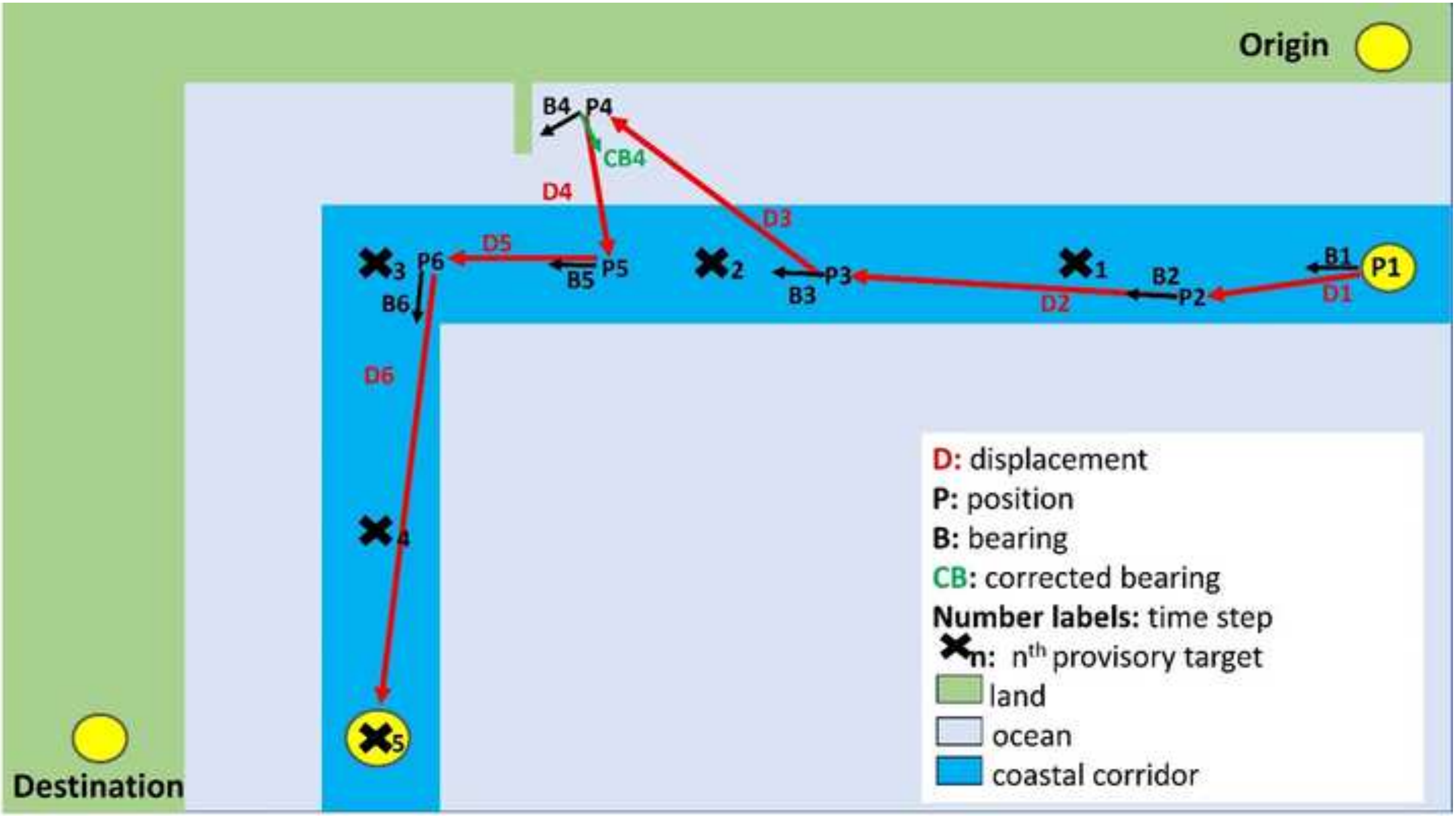

Click here to access/download;Figure;Fig21.tiff

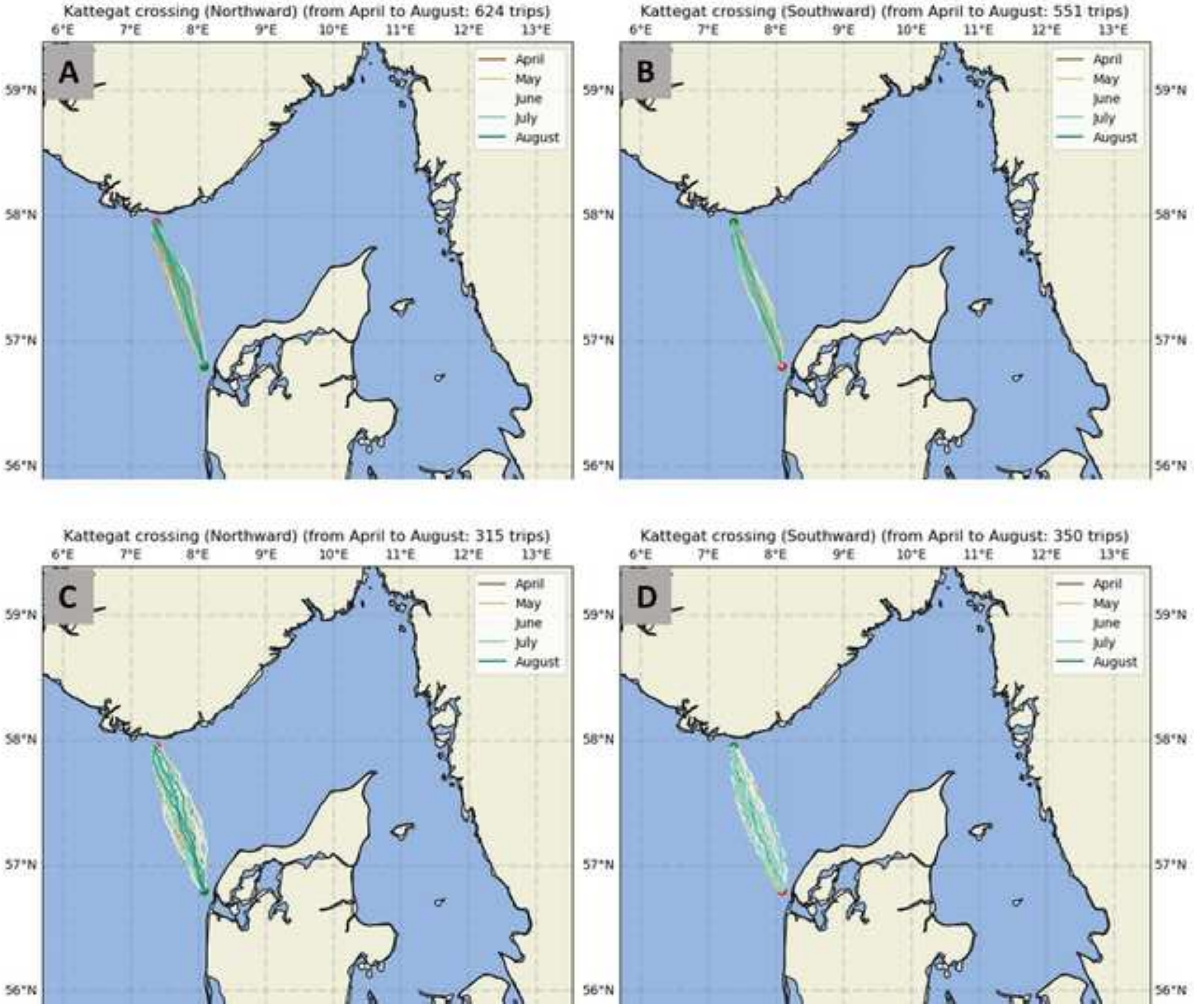

Click here to access/download;Figure;Fig22.tiff

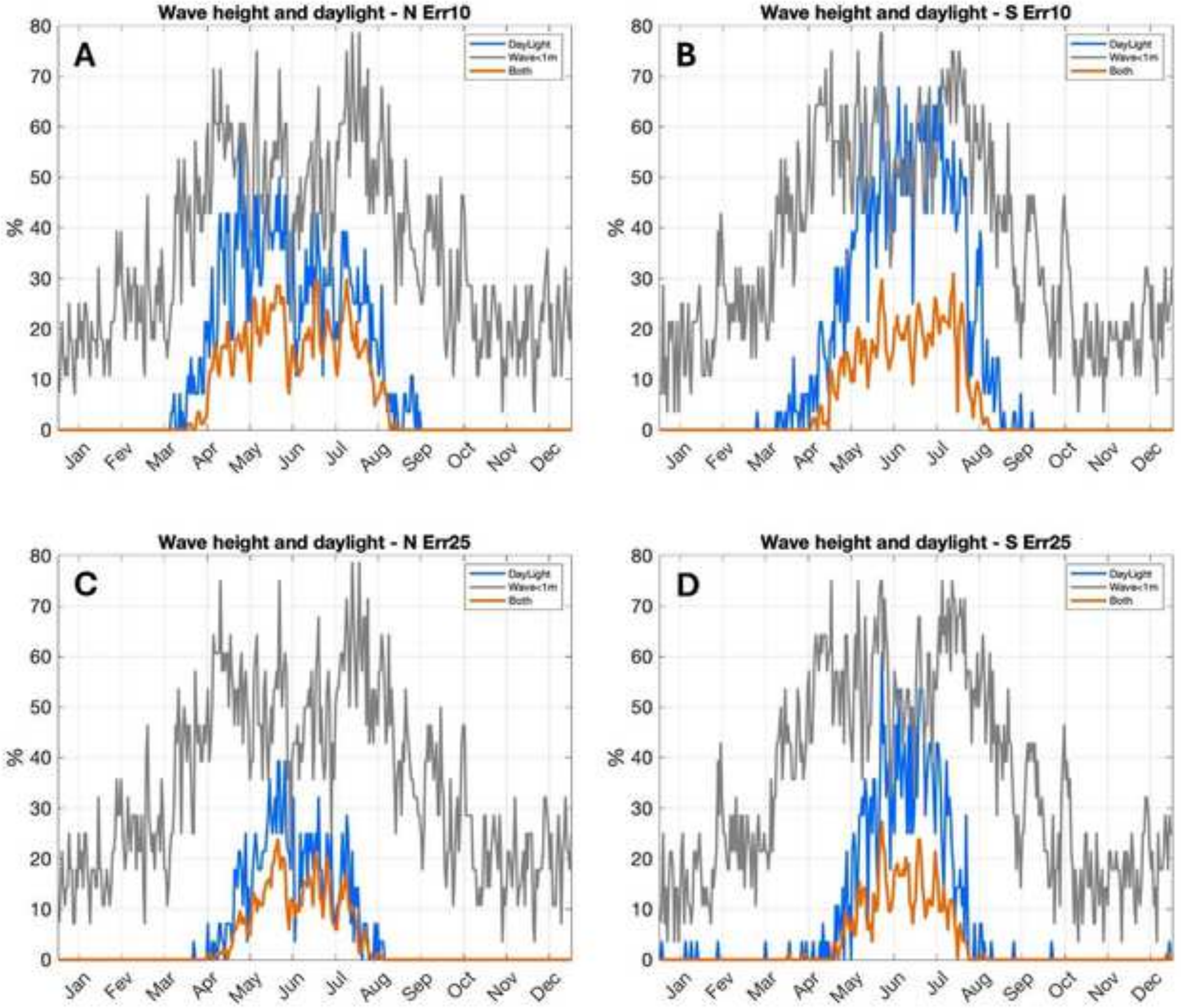

Click here to access/download;Figure;Fig23.tiff

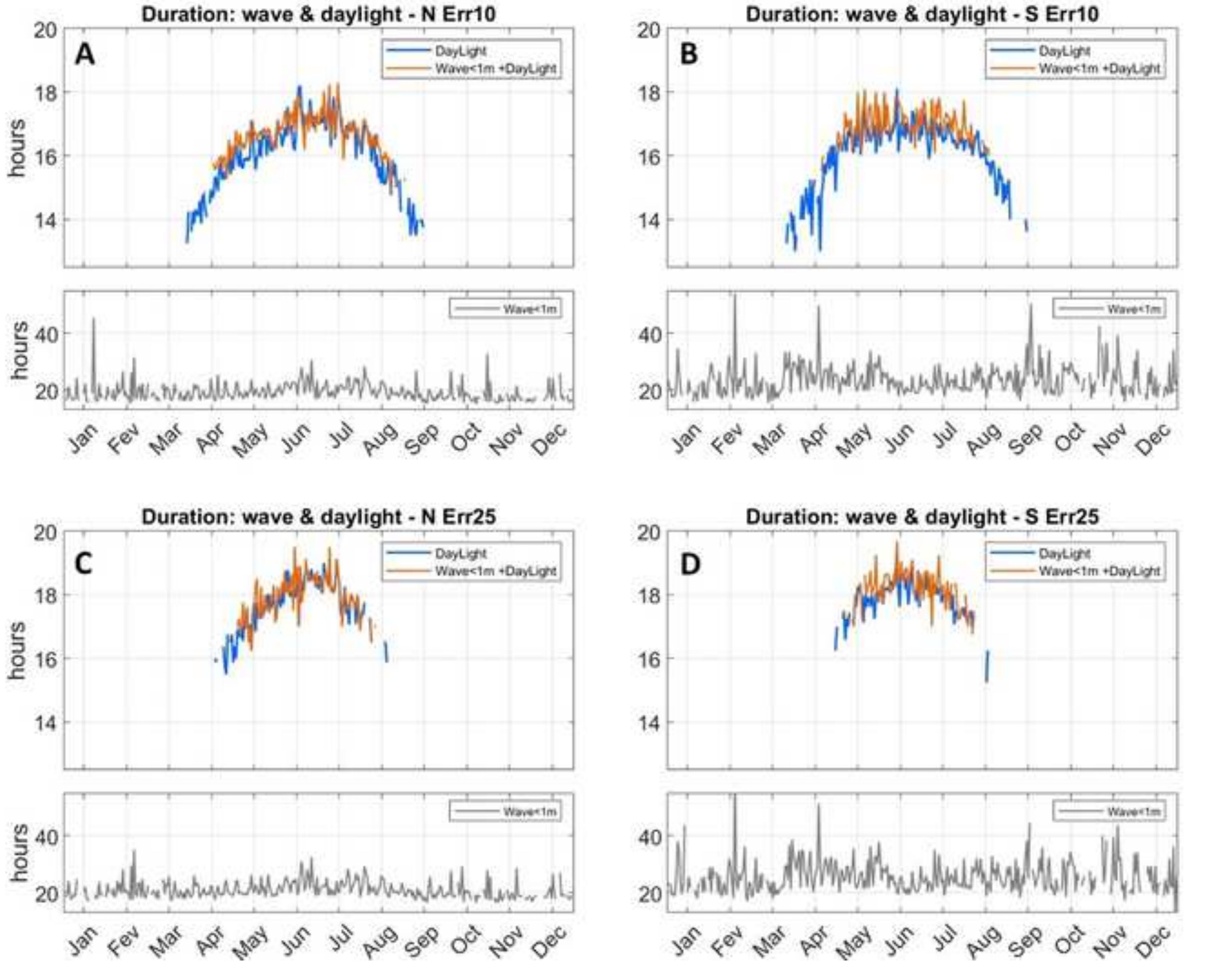

Click here to access/download;Figure;Fig24.tiff

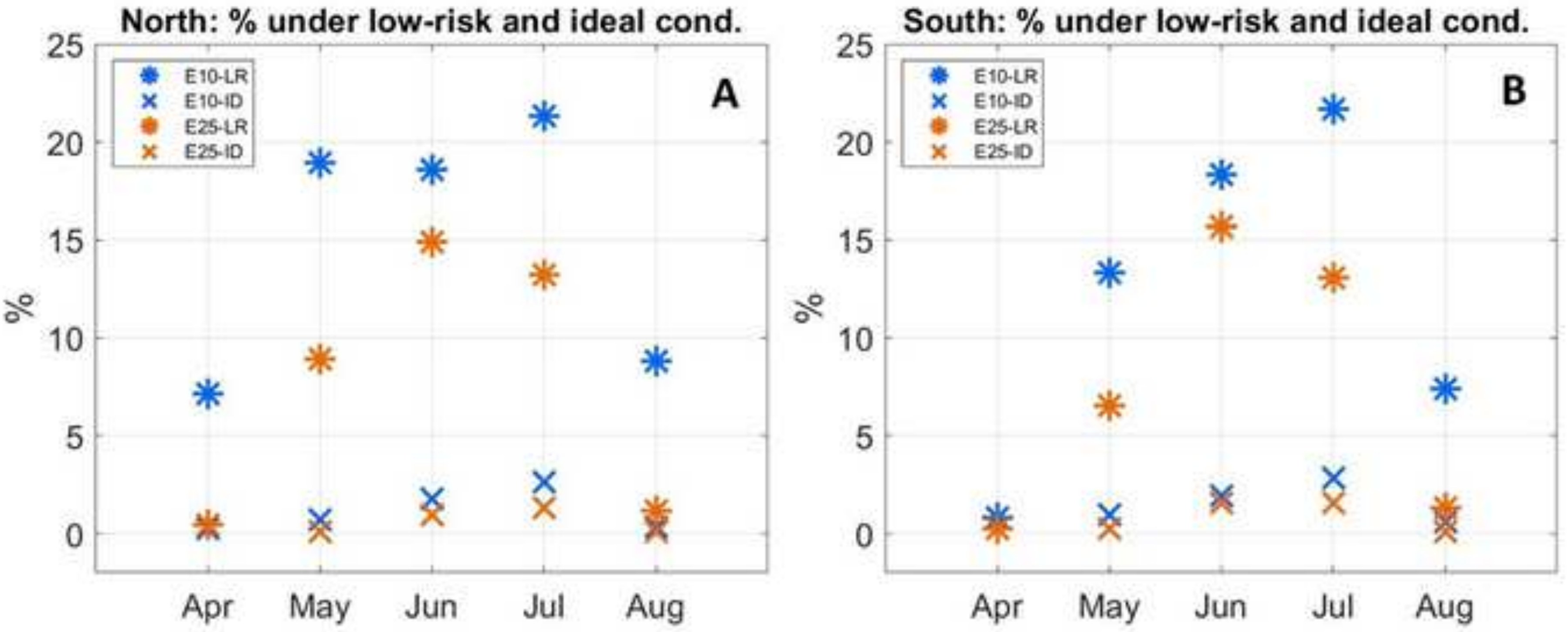

Click here to access/download;Figure;Fig25.tiff

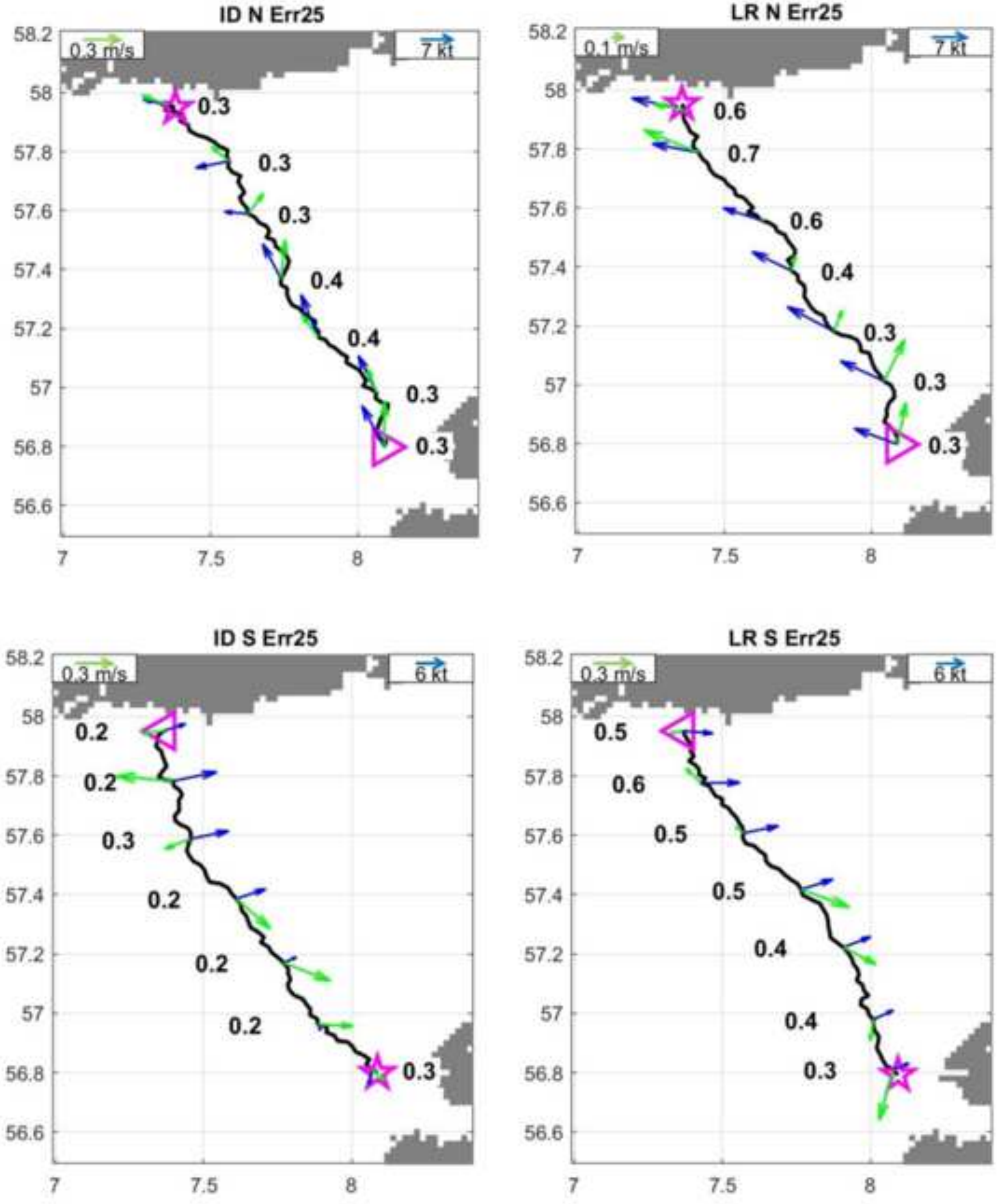

Click here to access/download;Figure;Fig26.tiff

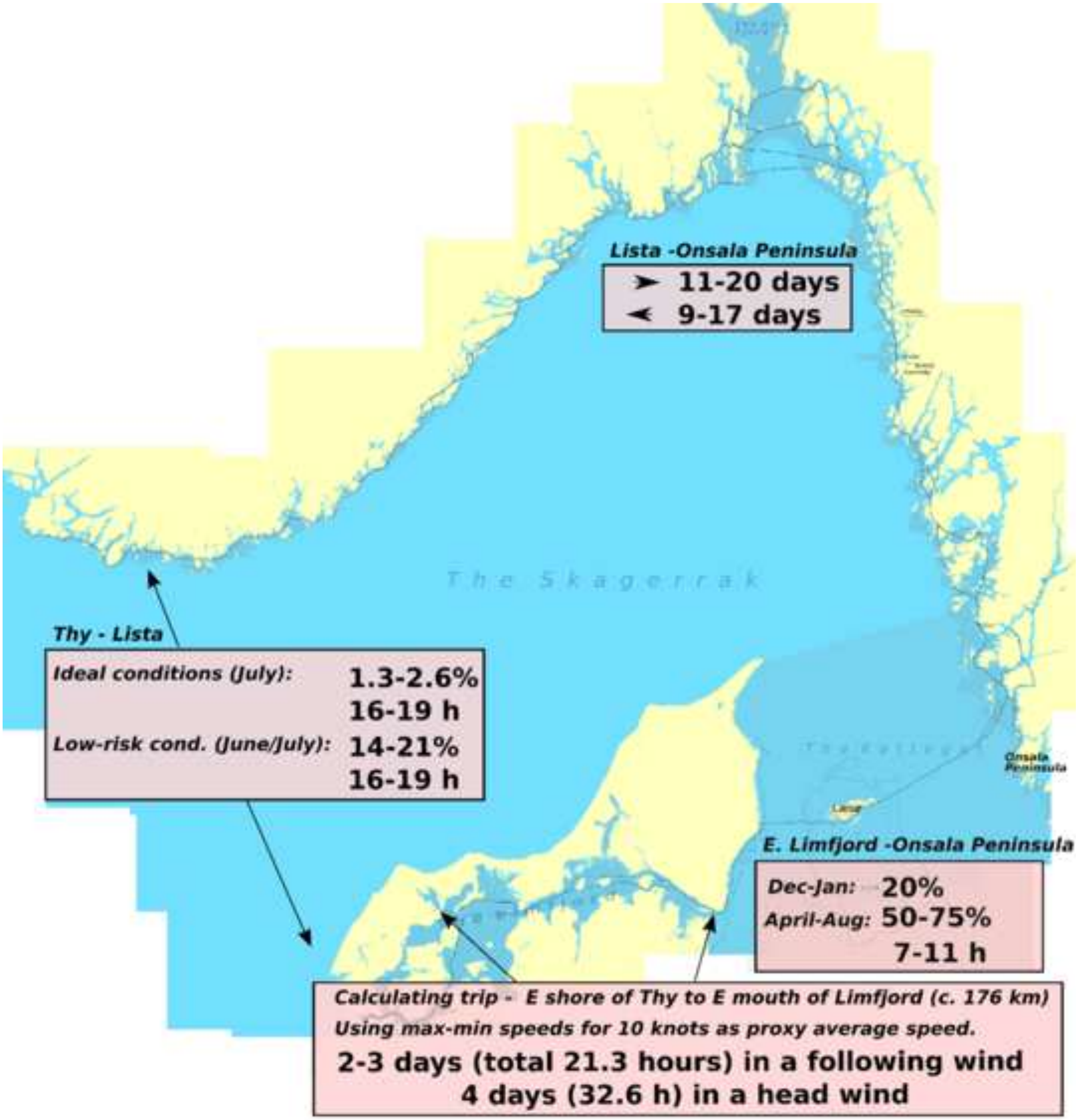

Click here to access/download;Figure;Fig27.tiff

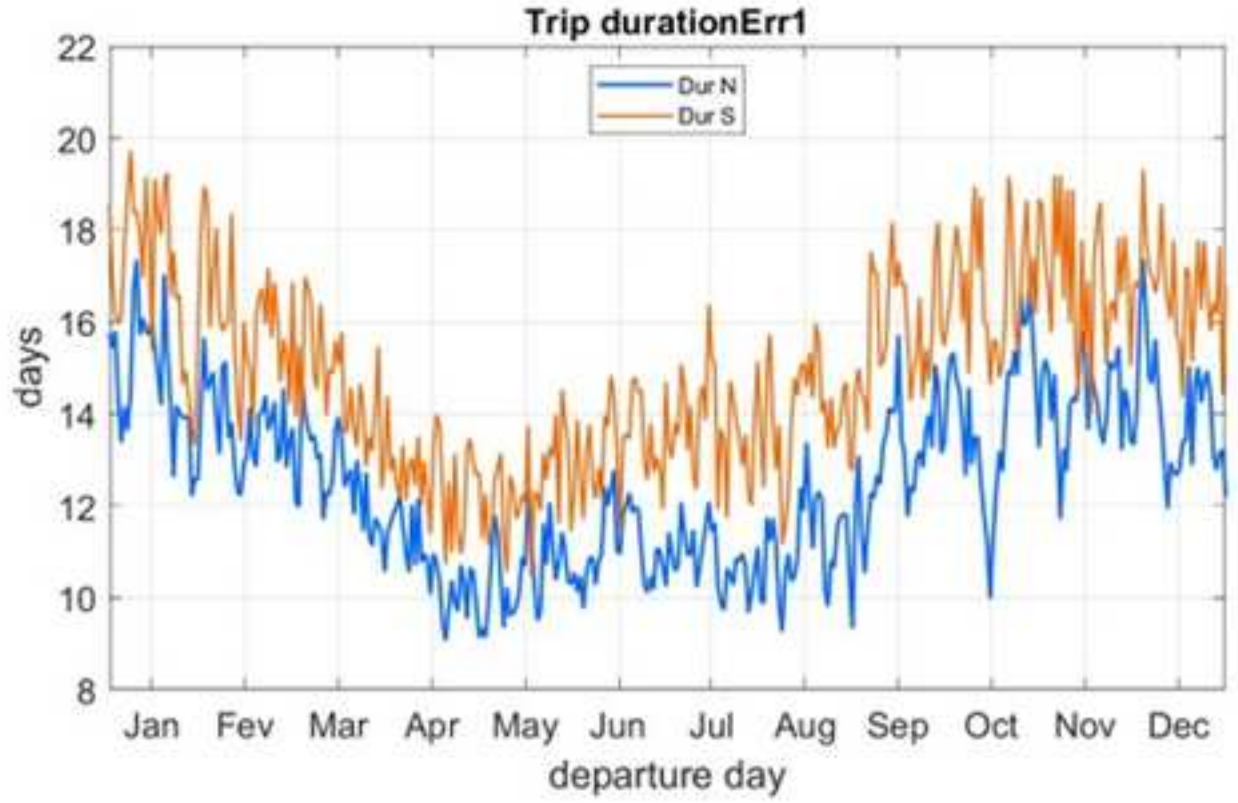

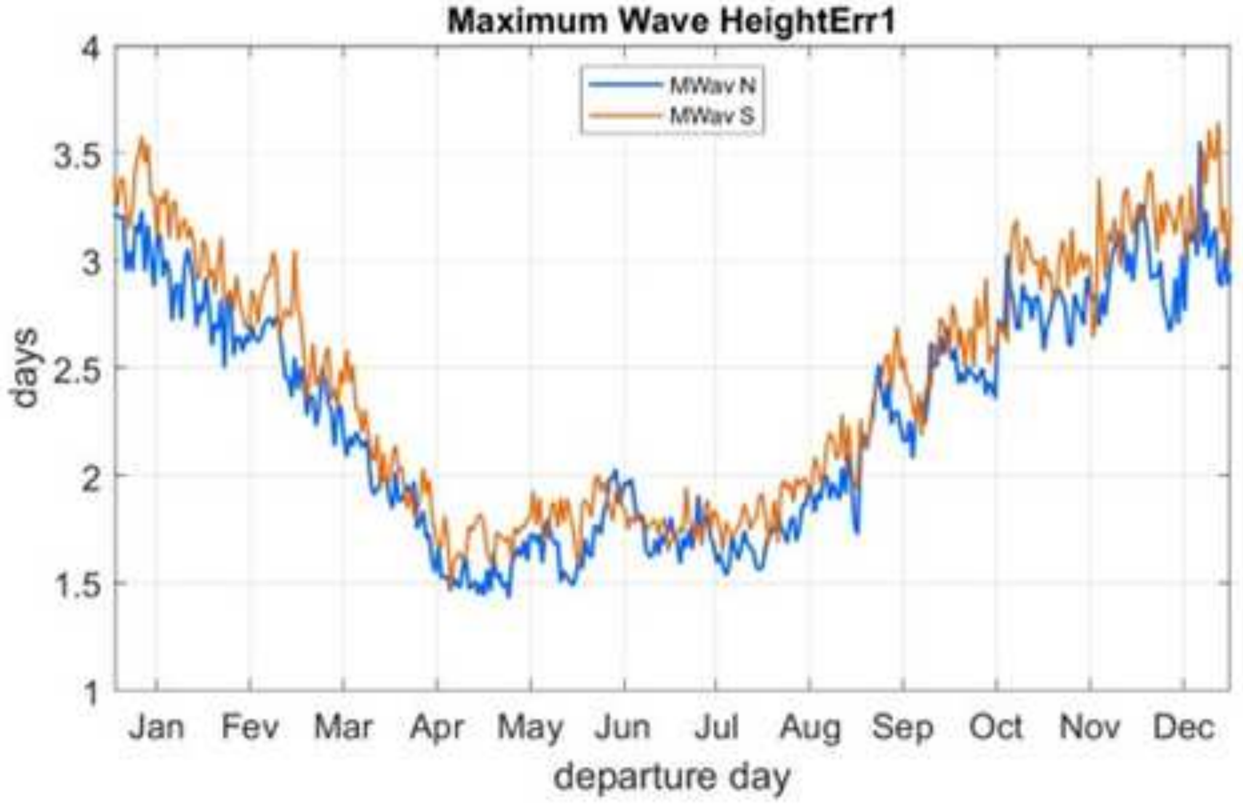

Click here to access/download;Figure;Fig28.tiff

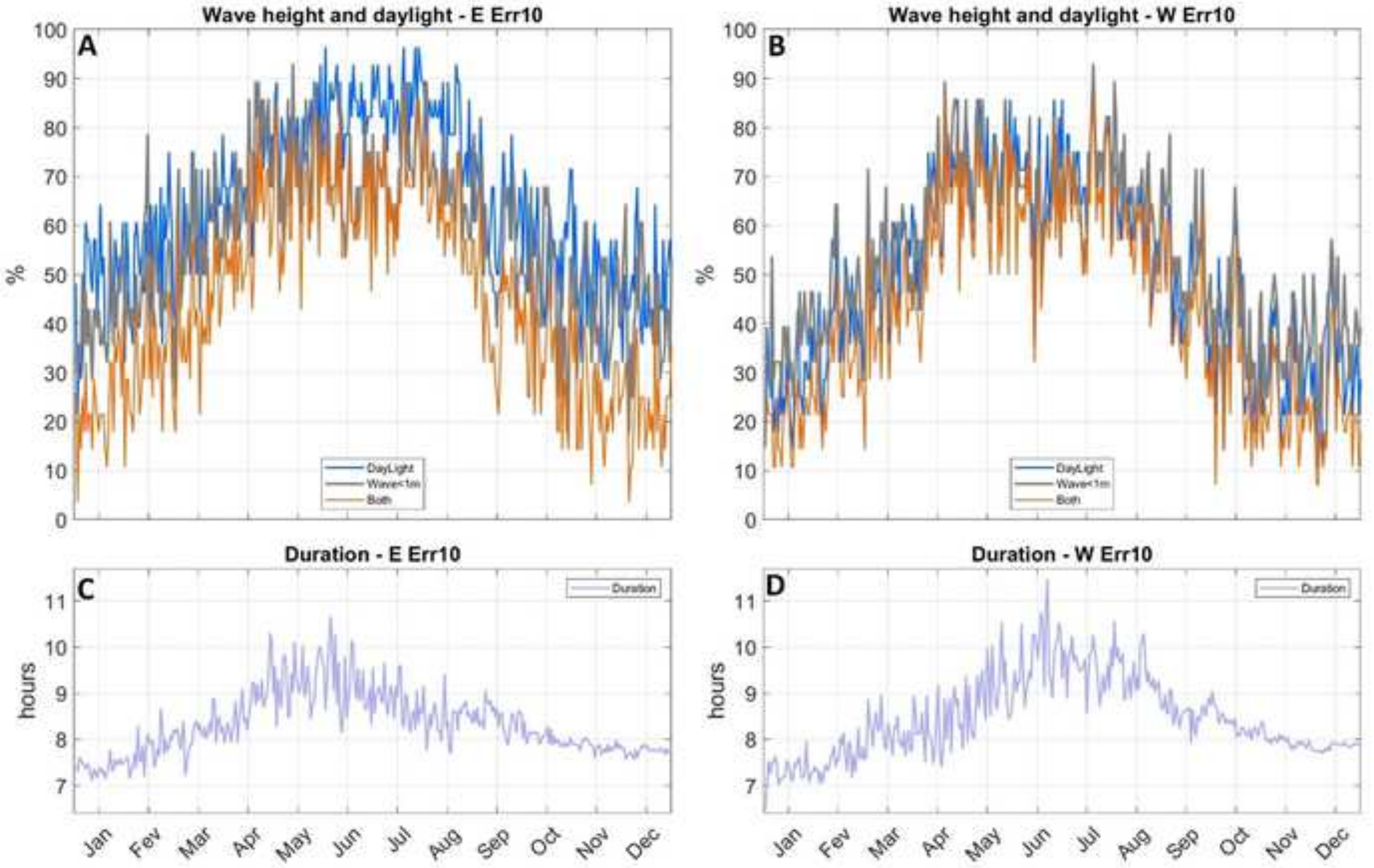

Click here to access/download;Figure;Fig29.tiff

Hjortspring - Calculations for total displacement long distance paddling (18 h).

Food & water

8000-10000 calories (kcal) per day per person
6 litre water/person/day x 22 = 132 kg

Food and water = c. 156.4 kg per day. 3 days = 469 kg

Boat & basic equipment

Boat, paddles, steering oars, bailer = 530 kg
Anchor/sea anchor, 3? 50 kg each with ropes? 150 kg
Tent, cooking pot = 30 kg

Crew and personal affects

Crew weight 65 kg x 22 = 1430 kg
Clothes/sea clothes, blanket, weapon 25 kg each = 550 kg

Total: 2846 kg. Calculated max displacement c. 3000 kg

**1 day food provisions only! Little room for cargo.
Every kg counts! Can carry c. 154 kg**

Click here to access/download;Figure;Fig30.tiff

Hjortspring - Calculations for total displacement long distance paddling (10 h/d).

Food & water

5000-8000 calories (kcal) per day per person
4.5 litre water/person/day x 22 = 99 kg

Food and water = c. 120 kg per day. 3 days = 360 kg

Boat & basic equipment

Boat, paddles, steering oars, bailer = 530 kg
Anchor/sea anchor, 3? 50 kg each with ropes? 150 kg
Tent, cooking pot = 30 kg

Crew and personal affects

Crew weight 65 kg x 22 = 1430 kg
Clothes/sea clothes, blanket, weapon 25 kg each = 550 kg

Total: 2810 kg. Calculated max displacement c. 3000 kg

1 day food provisions only 190 kg left for cargo!
Every kg counts! If 2 days provisions, only 70 kg...
