# Supplementary material for: Seafaring and navigation in the Nordic Bronze Age: The application of an ocean voyage tool and boat performance data for comparing direct open water crossings with sheltered coastal routes
Source: PLoS One. 2025 Apr 2;20(4):e0320791. doi: 10.1371/journal.pone.0320791 (PMC11964266; doi:10.1371/journal.pone.0320791)
Supplement: Attachment — Submitted filename: PLOS_review.docx [file pone.0320791.s001.docx]

**Overall Summary**

This paper presents a fascinating application of computer simulations to model Bronze Age seafaring between Denmark and Norway. While the focus is on a specific region and time period, the methodology has significant potential for broader application by seafaring modelers, ethnographers, and experimental archaeologists working in similar contexts. It is a shame then that the authors make a claim that maritime activities “are most often illustrated merely by the addition of large arrows on a map.” Sadly, this overlooks a well-developed and sophisticated body of work already engaging in seafaring modelling. While the paper proposes novel approaches, it misses an opportunity to build on this existing corpus. Acknowledging and situating the study within the broader context of previous research would strengthen its contribution.

There is a variety of work that has been conducted towards modelling actual seafaring routes through more blended perspectives as proposes by the authors. These are some references that could have been considered:

Callaghan, Richard, and Chris Scarre. (2009) "Simulating the western seaways." *Oxford Journal of Archaeology* 28.4: 357-372.

Davies, B., Bickler, S. H., & Traviglia, A. (2015). Sailing the simulated seas: A new simulation for evaluating prehistoric seafaring. In *Across Space and Time: Papers from the 41st Conference on Computer Applications and Quantitative Methods in Archaeology, Perth, 25–28 March 2013* (pp. 215-223). Amsterdam: Amsterdam University Press.

Indruszewski, George, and C. Michael Barton. (2008) "Cost surface DEM modeling of Viking Age seafaring in the Baltic Sea." *Beyond Illustration: 2D and 3D Digital Technologies as Tools for Discovery in Archaeology, International Series*, 56-64.

Knappett, Carl, Tim Evans, and Ray Rivers. (2008) "Modelling maritime interaction in the Aegean Bronze Age." *Antiquity* 82.318: 1009-1024.

Safadi, Crystal. (2016) "Wind and wave modelling for the evaluation of the maritime accessibility and protection afforded by ancient harbours." *Journal of Archaeological Science: Reports* 5: 348-360.

Sanmark, A., & McLeod, S. (2024). Norse Navigation in the Northern Isles. *Journal of the North Atlantic*, *2024*(44), 1-26.

*Also, see Ward et al 2016 for an interesting read on the impact of GIA on past tides and Blankshein 2022 for incorporation of palaeotidal models into seafaring models.

Ward, Sophie L., et al. "Sensitivity of palaeotidal models of the northwest European shelf seas to glacial isostatic adjustment since the Last Glacial Maximum." *Quaternary Science Reviews* 151 (2016): 198-211.

Blankshein, S. L. (2022). (Sea) ways of perception: an integrated maritime-terrestrial approach to modelling prehistoric seafaring. *Journal of archaeological method and theory*, *29*(3), 723-761.

If the authors take this previous work into consideration and include or at least highlight the broader applicability of this research, the paper’s relevance would be furthered and this work could more effectively contribute to the corpus of research to which it belongs.

**Methodology**

The study’s approach, combining vessel and crew performance data with environmental variables in simulations, is particularly interesting. The use of data derived from trials on a reconstructed Bronze Age vessel is exemplary and contributes significantly to the paper's credibility. The parameters used and the detailed outputs provided are clear and have the potential to be well-illustrated.

However, some methodological limitations need addressing:

- Software Transparency: The lack of detail about the simulation tool used hinders the replicability of the study. While the detail given for the inputs and results partially mitigate this issue, providing some insight into the software used and its functionality would allow other researchers to apply or adapt the methodology. This is especially important if expanding upon the significance of this method. Furthermore, knowing the software used (its parameters, limitations, etc.) would enable greater appreciation for or at least a better understanding of the results.
- "Provisory Targets" for Coastal Routes: Modelling coastal navigation using fixed targets restricts the exploration of alternative routes that may bypass certain sections of coast due to geographic, environmental, or physiological factors. A more flexible approach—such as applying a coastal buffer within which the models can operate—might provide a more realistic representation of navigational strategies. This would allow the simulations to explore alternative routes along the coast and perhaps skip sections of coast where it is quicker to move across a stretch of water than it is to move more tightly along the coast (similar to the various routes in Fig 10, inset C). However, this is seen as a minor limitation.
- Tidal Cycles: Also, taking the monthly average of sea conditions, while admirable in trying to reach both larger (annual) and smaller (monthly) scales within the simulations, excludes critical short-term cycles like tides. Tides can have a significant impact on bays, sounds, fjords, or estuaries, especially in narrow channels like those often found along the Atlantic façade of Europe. In some cases tides can create currents or even eddies that challenge even wind induced currents – e.g. the ebb of a spring tide through a narrow channel can be quite strong and in some sea lochs in Scotland have huge impacts on the ability to move through these regions using paddle propulsion alone (Blankshein et al 2022; Ward et al 2016; McGrail). Even basic assumptions, such as favouring neap tides or considering spring tides in specific contexts, could improve the granularity and realism of the simulations. Regardless, this omission of tides should at least be acknowledged, or preferably justified by your specific considerations, environmental conditions, aims and assumptions. Again, this is seen as a minor limitation but one that should at least be addressed in a few sentences.

Overall, the method is interesting and contributes to the study of both ancient seafaring more broadly and Bronze Age seafaring in Scandinavia more specifically. However, clarification of the simulation software, input data types, resolutions and sources, would further contextualize the results and justify certain omissions.

**Results/Conclusions**

The results raise valuable questions rather than offering definitive answers, which is appropriate given the speculative nature of simulation-based studies. However, some areas warrant further exploration:

Archaeological Evidence: The discussion would benefit from linking the simulated route along the coastal route to any archaeological finds – if any exist (there are 3 stars indicated in Fig 10 but I can’t read the text). Evidence of trade or activity could substantiate claims about the feasibility or popularity of specific routes (eg, you mention place names and portages but without any detail); likewise, an absence of evidence could support the claim for shorter open sea voyages. Regardless, a discussion of this evidence (whether it exists or not) would lend to a more nuanced discussion of the results and your interpretation of them, as discussed below.

Route Diversity: The possibility of different routes being used for distinct purposes or at varying times of the year is compelling. For example, speedier direct crossings might serve cargo transport, while the coastal route could offer safer or more leisurely alternatives. Exploring this dynamic in greater detail would enhance the paper’s interpretive depth.

Vessel Functionality: The suggestion that the Hjortspring boat is unsuitable for cargo and optimized for speed aligns well with the hypothesis of route variability. Highlighting how vessel design aligns with navigational choices would add nuance to the conclusions.

The speculative nature of simulation studies is acknowledged, but the authors should emphasize how these results generate new avenues of inquiry rather than definitive conclusions. Is there any evidence of trade or activity in these regions at all?

**Grammar, spelling, etc.**

While the content is strong, the paper requires significant editorial work:

Figures:

Many figures are of poor resolution, making symbols and labels illegible (eg, Figures 2 and 3). Figures should be revised to ensure clarity and readability, with high-resolution exports suitable for publication.

Also, the figure numbers found with the captions are inconsistent throughout the text, which was confusing for the reviewer.

Proofreading:

Numerous typographical errors (e.g., missing conjunctions, duplicated words) and overly long sentences detract from the paper’s clarity. Some of these have been marked in the text but there are others. Between this and the figure captions, this paper could benefit from a thorough revision and final proofing before publication.

**Recommendation**

The study presents a promising contribution to seafaring archaeology but requires revisions before publication. Addressing the methodological concerns, situating the work within existing literature, and improving the presentation would significantly enhance its impact and reach.
