# Supplementary material for: Seafaring and navigation in the Nordic Bronze Age: The application of an ocean voyage tool and boat performance data for comparing direct open water crossings with sheltered coastal routes
Source: PLoS One. 2025 Apr 2;20(4):e0320791. doi: 10.1371/journal.pone.0320791 (PMC11964266; doi:10.1371/journal.pone.0320791)
Supplement: Attachment — Submitted filename: Response to Reviewers.docx [file pone.0320791.s004.docx]

**Response to reviewers:**

**Thank you for asking us to submit a revised version of the original manuscript titled:**

**‘Seafaring and navigation in the Nordic Bronze Age. Comparing direct open water crossings with sheltered coastal routes using an ocean voyage simulation tool’**

**Answers to Journal Requirements.**

1.Endeavour to meet style requirements, including file naming.

2. There was no additional external funding received for this study. The statement has been amended to include ‘all’ the funding or sources of support etc. etc.

3.We have removed ‘ this work is supported by…’ from Acknowledgements and are happy with the phrase ‘ this work is supported by Riksbankens etc. etc.’

4. Instead of providing trajectories via contact name and email, all simulations and trajectories are now freely available at <https://github.com/mtomasini/PLOS-Voyager>

5. The referenced unpublished material by Scheen, R, has been replaced with a reference to published material. This has been addressed in the manuscript.

6. Original copyright holder of Figures 1,2,3,4,8,10,11 and 26. These maps have been made by extrapolating data from SGU ([https://www.sgu.se/produkter-och-tjanster/kartor/kartvisaren/jordkartvisare/strandforskjutningsmodell/](https://www.sgu.se/produkter-och-tjanster/kartor/kartvisaren/jordkartvisare/strandforskjutningsmodell/)), using a model by Påsse and Daniels 2015. The maps have been manipulated and created by Ashely Green and Boel Bengtsson, using ArcGIS Pro and Inkscape. Thus original copyright holders are Ashely and Bengtsson. In figure 3, two landscape drawings are included. Although in both cases the artists have been deceased for over 100 years, we have gained specific permission from Kristian Jensen, editor at Jysk Arkæologisk Selskab (this email will be uploaded for you to view). The other drawing here, showing Bulbjerg, is collected from an open source (<https://commons.wikimedia.org/wiki/File:Bulbjerg-Skarreklit_JTH.jpg?uselang=en#Licensing>), stating it is under no copyright restrictions for use.

7. Figure 5 is a photo taken by Boel Bengtsson (myself). I allow it to be published under the CC BY 4.0 license. Likewise, the photo in Fig 12 is taken by myself.

8. All figures will be updated and resubmitted ensuring a high level of detail is retained.

**Overall Summary**

This paper presents a fascinating application of computer simulations to model Bronze Age seafaring between Denmark and Norway. While the focus is on a specific region and time period, the methodology has significant potential for broader application by seafaring modelers, ethnographers, and experimental archaeologists working in similar contexts.

In response, we have shifted focus onto the application of the ocean modelling tool for evaluating Nordic Bronze Age Seafaring.

Therefore, we have altered the title to

**Seafaring and navigation in the Nordic Bronze Age. The application of an ocean voyage tool and boat performance data for comparing direct open water crossings with sheltered coastal routes.**

And the abstract to:

This study presents an “ocean voyaging tool” that combines predicted vessel performance data with agent-based simulations. This tool offers a new way to assess navigation and seafaring abilities in prehistory while also enabling the direct comparison between different type vessels and vessel configuration, navigational skills and propulsion. Results are filtered using certain limitations on safety (wind strength, wave height, light etc.) and navigational error. The method is here used to compare direct open water (c. 110 km) and coast hugging (c. 700 km) voyages between Jutland and south-west Norway in the Early Nordic Bronze Age, two areas that were closely connected from the Late Neolithic throughout the Bronze Age (c. 2100-1500 BC). Simulated results suggest that although the longer coastal route is usable all year round, direct open sea voyages, which included navigation out-of-sight-of-land for up to 50 km, were most likely undertaken. Such voyages would have necessitated boats capable of withstanding and maintaining directional control in ≤ 1 m waves and winds of up to 10 knots (5 m/s) at a minimum. Furthermore, these simulations highlight the comparable advantage of sail over paddling for transporting cargo over long distances (journeys of more than one day’s length).

It is a shame then that the authors make a claim that maritime activities “are most often illustrated merely by the addition of large arrows on a map.” Sadly, this overlooks a well-developed and sophisticated body of work already engaging in seafaring modelling. While the paper proposes novel approaches, it misses an opportunity to build on this existing corpus. Acknowledging and situating the study within the broader context of previous research would strengthen its contribution.

There is a variety of work that has been conducted towards modelling actual seafaring routes through more blended perspectives as proposes by the authors. These are some references that could have been considered:

Callaghan, Richard, and Chris Scarre. (2009) "Simulating the western seaways." *Oxford Journal of Archaeology* 28.4: 357-372.

Davies, B., Bickler, S. H., & Traviglia, A. (2015). Sailing the simulated seas: A new simulation for evaluating prehistoric seafaring. In *Across Space and Time: Papers from the 41st Conference on Computer Applications and Quantitative Methods in Archaeology, Perth, 25–28 March 2013* (pp. 215-223). Amsterdam: Amsterdam University Press.

Indruszewski, George, and C. Michael Barton. (2008) "Cost surface DEM modeling of Viking Age seafaring in the Baltic Sea." *Beyond Illustration: 2D and 3D Digital Technologies as Tools for Discovery in Archaeology, International Series*, 56-64.

Knappett, Carl, Tim Evans, and Ray Rivers. (2008) "Modelling maritime interaction in the Aegean Bronze Age." *Antiquity* 82.318: 1009-1024.

Safadi, Crystal. (2016) "Wind and wave modelling for the evaluation of the maritime accessibility and protection afforded by ancient harbours." *Journal of Archaeological Science: Reports* 5: 348-360.

Sanmark, A., & McLeod, S. (2024). Norse Navigation in the Northern Isles. *Journal of the North Atlantic*, *2024*(44), 1-26.

*Also, see Ward et al 2016 for an interesting read on the impact of GIA on past tides and Blankshein 2022 for incorporation of palaeotidal models into seafaring models.

Ward, Sophie L., et al. "Sensitivity of palaeotidal models of the northwest European shelf seas to glacial isostatic adjustment since the Last Glacial Maximum." *Quaternary Science Reviews* 151 (2016): 198-211.

Blankshein, S. L. (2022). (Sea) ways of perception: an integrated maritime-terrestrial approach to modelling prehistoric seafaring. *Journal of archaeological method and theory*, *29*(3), 723-761.

If the authors take this previous work into consideration and include or at least highlight the broader applicability of this research, the paper’s relevance would be furthered and this work could more effectively contribute to the corpus of research to which it belongs.

Response. We have tried to address this by changing/inserting the following:

*Yet, the boats, the navigational skills and the actual sea routes that undoubtedly underpinned such occurrences remain elusive and the complex systems integrating these activities are most often illustrated merely by the addition of large arrows on a map.*

*Simulations of seafaring voyages as method to understand maritime mobility in prehistory were first developed in the 1970s with a view to exploring whether the Polynesian expansion could have happened by chance, that is, through passive voyages where boats were simply drifting with winds and currents (Levison et al. 1973). More recent simulations focus on passive drift scenarios, sea journeys based on very rough estimates of paddling speed or the simulation of historical voyages where there are detailed diaries of passage and course (see e.g. Callaghan1999, 2001, 2003; Callaghan & Scarre 2009; D’Cunha et al. 2021; Fitzpatrick & Callaghan, 2008; Montenegro et al. 2016; Temmen et al. 2022). Other efforts to break down and isolate some of the multiple variables needed to understand prehistoric seafaring, include the application of least cost models, where winds, currents and topography information is used to generate routes that minimize traveling “costs”, such as duration or risk (see e.g. Indruszewski & Barton 2008; Blankshein 2022).*

*Meanwhile, there have been enormous improvements in digital hardware and software as well as in the resolution of the wind, currents, and other environmental data simulations are dependent on. For example, several ocean voyaging optimization software products on the market developed for use within the commercial shipping industry and amongst the boat racing community (see for example DECARTES & ADRENA). Attempts to apply aspects of such optimization models, using e.g. weather routing linked to land visibility and the detection of specific landmarks, has also recently been used as a way to explore prehistoric sea routes (Smith 2020). However, none of these applications allow for agency, that is seafarers making active choices based on specific conditions and their knowledge of their boat. For this, reliable boat performance data is needed (Callaghan 2001).*

Additional references here include:

Levison, M., Ward, R.G., and Webb, J. W., 1973. Settlement of Polynesia: A Computer Simulation. University of Minnesota Press. Minneapolis.

Callaghan, R. T., 1999. Computer Simulations of Ancient Voyaging. The Northern Mariner/Le Marin du nord, IX (2):11-22.

Callaghan, R. T., 2001. Ceramic Age Seafaring and Interaction Potential in the Antilles: A Computer Simulation. Current Anthropology. Volume 42(2):308-313.

Callaghan R. T., & Scarre C., 2009. Simulating the western seaways. Oxford Journal of Archaeology. 28(4): 357-372.

D’Cunha, M.G.T, A. Montenegro and J. Field, 2021. [Modeling water crossings leading to the arrival of early Homo in Sulawesi, Indonesia via paleoclimate drift experiments](https://doi.org/10.1016/j.jasrep.2021.103194), Journal of Archaeological Sciences: Reports 40

Fitzpatrick, S., M. & Callaghan R., 2008. Magellan's Crossing of the Pacific: Using Computer Simulations to Examine Oceanographic Effects on One of the World's Greatest Voyages. The Journal of Pacific History 43(2): 145-165.

Montenegro, A., R. Callaghan and S.M. Fitzpatrick, 2016. [Using Seafaring Simulations and Shortest Hop Trajectories to Model the Prehistoric Colonization of Remote Oceania](https://www.pnas.org/doi/full/10.1073/pnas.1612426113), 2016, Proceedings of the National Academy of Sciences, 113, 12685-12690

Temmen, J.,A. Montenegro, S. Juras, J. Field and J. DeGrand, 2022. Floating the Sweet Potato to Polynesia:Considering the feasibility of oceanic drift for the prehistoric introduction of the sweet potato (Ipomoea batatas) to Pacific Islands. Quaternary Sciences Reviews, 295.

Smith, K., 2020. Modelling Seafaring in Iron Age Atlantic Europe. PhD. Kellogg College, University of Oxford.

**Methodology**

The study’s approach, combining vessel and crew performance data with environmental variables in simulations, is particularly interesting. The use of data derived from trials on a reconstructed Bronze Age vessel is exemplary and contributes significantly to the paper's credibility. The parameters used and the detailed outputs provided are clear and have the potential to be well-illustrated.

However, some methodological limitations need addressing:

- Software Transparency: The lack of detail about the simulation tool used hinders the replicability of the study. While the detail given for the inputs and results partially mitigate this issue, providing some insight into the software used and its functionality would allow other researchers to apply or adapt the methodology. This is especially important if expanding upon the significance of this method. Furthermore, knowing the software used (its parameters, limitations, etc.) would enable greater appreciation for or at least a better understanding of the results.

Response: We appreciate the reviewers concern with reproducibility and, following Plos One’s policy have uploaded the simulations code as follows:

*The input currents and wave data to run the simulations is obtainable from the Copernicus Marine Service (product DOIs:* [*10.48670/moi-00013*](https://doi.org/10.48670/moi-00013)*,* [*10.48670/moi-00014*](https://doi.org/10.48670/moi-00014)*,* [*10.48670/moi-00059*](https://doi.org/10.48670/moi-00059) *and* [*10.48670/moi-00060*](https://doi.org/10.48670/moi-00060)*); the input wind data are obtainable from the Copernicus regional reanalysis for Europe (CERRA) website (DOI:* [*10.24381/cds.622a565a*](https://doi.org/10.24381/cds.622a565a)*). The code for the simulations, as well as the aggregate of the results and trajectories are obtainable on Github* at <https://github.com/mtomasini/PLOS-Voyager>*.*

- "Provisory Targets" for Coastal Routes: Modelling coastal navigation using fixed targets restricts the exploration of alternative routes that may bypass certain sections of coast due to geographic, environmental, or physiological factors. A more flexible approach—such as applying a coastal buffer within which the models can operate—might provide a more realistic representation of navigational strategies. This would allow the simulations to explore alternative routes along the coast and perhaps skip sections of coast where it is quicker to move across a stretch of water than it is to move more tightly along the coast (similar to the various routes in Fig 10, inset C). However, this is seen as a minor limitation.

Response: The reviewer is correct. The use of sequential targets does not allow for exploration of alternate routes. We have added the following to the main text calling attention to this limitation:

*The adoption of sequential targets provides a trustworthy first order representation of coastal displacement but may overestimate trip duration by forcing boats to follow the coast in situations where navigators might be able to paddle or sail across the mouth of an embayment instead of following along its coast. In spite of this limitation, we believe sequential targets, given the lack of knowledge of Bronze Age navigation strategies and the large difference in duration between open ocean and coastal trips – is a viable option given our present goals.*

Tidal Cycles: Also, taking the monthly average of sea conditions, while admirable in trying to reach both larger (annual) and smaller (monthly) scales within the simulations, excludes critical short-term cycles like tides. Tides can have a significant impact on bays, sounds, fjords, or estuaries, especially in narrow channels like those often found along the Atlantic façade of Europe. In some cases tides can create currents or even eddies that challenge even wind induced currents – e.g. the ebb of a spring tide through a narrow channel can be quite strong and in some sea lochs in Scotland have huge impacts on the ability to move through these regions using paddle propulsion alone (Blankshein et al 2022; Ward et al 2016; McGrail). Even basic assumptions, such as favouring neap tides or considering spring tides in specific contexts, could improve the granularity and realism of the simulations. Regardless, this omission of tides should at least be acknowledged, or preferably justified by your specific considerations, environmental conditions, aims and assumptions. Again, this is seen as a minor limitation but one that should at least be addressed in a few sentences.

The reviewer makes a good point. We should have done a better job informing the reader about this. As well as clearly stating in the introduction that both **simulated routes are** **non-tidal**. The input data section now contains the following paragraph:

*Tidal flows, which can be significant in channels and near shore areas, are not present in the adopted current data. While tide should be considered when simulating particular trips being performed at specific dates and times, they play a much smaller role in affecting results in the type of analysis conducted here. Our vessels started/ended their displacements without any consideration for tidal phase. Assuming tidal currents were present in our input data, this would mean that among the more 10 thousand voyages simulated per experiment, trips would be initiated/ended under a large range of tidal amplitudes. Some trips would be started/ended under tidal currents in their favour, some with currents against them, other under weak tidal currents with little impact on vessel movement. If we were to average these tide-influenced results into the multi-year daily and monthly means presented below, the most significant tidal effects would be “averaged out”. More than that. Tides in the region are mostly semi-diurnal. In both daily coastal trips and open ocean crossings, vessels are in the water for multi-hour periods that last about 1 to almost 2 dominant tidal cycles. This means that, given their durations, even individual trips tend to experience tidal currents that both favour and hinder displacement toward a specific direction. In short, given how model output is treated and mean individual trip duration, the adoption of input currents that contained tidal flows would not have had a large impact on our conclusions.*

Overall, the method is interesting and contributes to the study of both ancient seafaring more broadly and Bronze Age seafaring in Scandinavia more specifically. However, clarification of the simulation software, input data types, resolutions and sources, would further contextualize the results and justify certain omissions.

Response: We are a bit confused by the reviewer’s request for clarification on data type, source and resolution. Could they be more specific? Our Environmental Data section provides what we believe are clear definitions of data type - regularly gridded ocean and atmosphere reanalysis, source – ECMWF Era5 and, resolution - all temporal and spatial resolutions are explicitly listed.

**Results/Conclusions**

The results raise valuable questions rather than offering definitive answers, which is appropriate given the speculative nature of simulation-based studies. However, some areas warrant further exploration:

Archaeological Evidence: The discussion would benefit from linking the simulated route along the coastal route to any archaeological finds – if any exist (there are 3 stars indicated in Fig 10 but I can’t read the text). Evidence of trade or activity could substantiate claims about the feasibility or popularity of specific routes (eg, you mention place names and portages but without any detail); likewise, an absence of evidence could support the claim for shorter open sea voyages. Regardless, a discussion of this evidence (whether it exists or not) would lend to a more nuanced discussion of the results and your interpretation of them, as discussed below.

Response. We have tried to make the evidence for contact clearer by rewriting and adding the following at the end of the archaeological background:

The strong connections between Thy and Lista in the Bronze Age is thus evidenced by the presence of imported lithics and metal artifacts from the Limfjord region, as well as notable similarities in burial practices in earthen mounds, cists made of stone slabs and similar architectural styles seen in three-aisled longhouses [4]. This similarity continuous west and northwards along the Norwegian coast. In contrast, when facing eastwards from the Lista peninsula the material and architectonic similarities are less clear. Burials are here instead mainly built with stones instead of earth (so called stone cairns), which appear in their thousands along the paleogeographic archipelagos of southern Norway and western Sweden, the majority of which are believed to date to the early Bronze Age (Claesson & Munkeberg 2004). An otherwise denominative feature for all southern Scandinavia is the rock art which appears in clusters along these coastal waterways and which date to c. 1700 – 200 BC. The maritime location of this imagery is further emphasized by its predominant focus on boats (Ling 2008; Bengtsson 2015, Bengtsson et al. 2024).

Bohuslän, placed roughly halfway along the coastal route between Lista and Thy, is the richest rock art area in Europe and in Scandinavia, featuring over 10,000 boat images, and is believed to have been an important boatbuilding and transit area in the Bronze Age (Kristiansen 1999, 89; Ling et al. 2024). Relatively large concentrations of flint and sickle daggers as well as bronzes found in this region points towards significant and often intense communication with the Jutland also for this region from the late Neolithic and throughout the Bronze Age (Apel 2001; Bengtsson 2017).

Thus, whereas rock art and general artefacts point towards the possibility that the two regions of Thy and Lista might have been communicating via the coastal route, the very specific similarities in certain types of materials and architectonic expressions might suggest the coastal route was bypassed altogether. So far, no studies have quantitatively modelled maritime travels using either route. Frode Kvalø[11] has done a thorough study on the possibilities of the direct routes across the Kattegat Strait based on oceanographic, ethnographic and experimental data., whereas e.g. Bengtsson (2015, 2017) instead has focused on potential seafaring routes between Bohuslän and Jutland, identifying e.g. the relative safety afforded to small open boats operating within archipelagos or along lee coast in relation to the direction and strength of the wind. However, there are now several new methodological and computational considerations that needs to be evaluated, and which might help shed new light on the nature of maritime communication between the two regions in the Bronze Age.

Claesson, P. & Munkenberg, B. (eds) 2004. Gravar och ritualer. Bohusläns museum, Kulturhistoriska dokumentationer 15. Uddevalla.

In response to a question from the review whether we have made a model for predicted sailing abilities, the answer is yes. However, we have chosen to only focus on the paddling model for this paper. To make this clear, we have added a line stating *This paper will focus on the paddling model*. Just above the heading Paddling model.

Route Diversity: The possibility of different routes being used for distinct purposes or at varying times of the year is compelling. For example, speedier direct crossings might serve cargo transport, while the coastal route could offer safer or more leisurely alternatives. Exploring this dynamic in greater detail would enhance the paper’s interpretive depth.

Response. This is one of many aspects that can be analysed in the future. We have further studies in the pipeline where this will be explored more in detail.

Vessel Functionality: The suggestion that the Hjortspring boat is unsuitable for cargo and optimized for speed aligns well with the hypothesis of route variability. Highlighting how vessel design aligns with navigational choices would add nuance to the conclusions.

Response, this is implied in the study already. In order to fully assess this we need more data on impact of waves on boat speed and crew endurance over long distances, which has been added to the conclusions. We have added in the discussion the following:

An interesting follow-on question here is what might have led to the establishment of a direct route between Thy and Lista and when such voyages might have commenced? Here a knowledge of the significance of thermal cumulus clouds forming over land is likely to have been crucial (Fig. 5). Such clouds can reach heights of up to 2000 meters or more when fully developed, usually between 5 to 20 kilometres inland from the coast (Fig. 31). During the right atmospheric conditions, such clouds forming over the Norwegian coast are therefore likely to be visible when looking north from Hanstholm, even when calculating on a cloud height of only 1000 meters, and when just standing on the beach if such clouds were fully developed. This would have providing firm directions of a distant landmass across the Skagerrak, meaning direct crossings might have been initiated from either direction (Thy or Lista) as and when seafarers were capable of such journeys and the needs for such journeys outweighed the perceived risks of undertaking them. The archaeological evidence suggests this happened already around 2300 BC. At this time archaeological evidence suggest the northward coast from Lista towards the Stavanger fjord was also regularly used, a comparatively exposed stretch of coast that is likely to have necessitated vessels of similar capabilities as those used for direct crossings of the Skagerrak [3].

**Fig 31. Showing the distance at which cumulus clouds can be detected during ideal atmospheric conditions. Calculations here are based on cloud formation reaching a height of 1000 meters. Cumulus clouds can build up to over 2000 meters height and would then have been visible by a person standing on the shore looking across without the need to stand at a height.**

Thus, the methods and tools presented here for evaluating prehistoric sea travels also allow for very fine-grained discussions on what the minimum boat requirements would have been for particular sea journeys.

The speculative nature of simulation studies is acknowledged, but the authors should emphasize how these results generate new avenues of inquiry rather than definitive conclusions. Is there any evidence of trade or activity in these regions at all?

Response. There is evidence of trade but apart from artefacts, rock art and stone cairns (and a few mounds), Bronze Age houses are scarce along the coastal route (most likely located under present day farmsteads, some of which are found near rock art sites). As such, this presents a problem. We used the age-old hypothesis of direct sea crossings between Thy and Lista to provide a limited study while developing the ocean voyaging tool.

What can this lead to

In response to this we have added.

Allowing for the interaction between predicted boat performance data within multiple years of environmental data makes it possible to break down and analyse all the multitude of components that underpin sea travel.

The model developed to predict boat performance of the *Tilia Alsie*, can be adapted to any type of prehistoric or modern vessel provided it’s hull shape is digitally recorded, and there are sufficient recorded parameters of the vessel. This calls for the systematic recording of all types of prehistoric boat reconstructions (Bengtsson in press), which includes the impact of waves on boat speed and crew endurance. Such information would allow for the direct comparison of results from different types of boats (prehistoric/modern whether e.g. paddled/sailed), encountering the same multi-year environmental data, while travelling between points within the same geographic time frame, or, corresponding geographic time frames where coastal configuration has changed. This offers huge potential for future studies of seafaring in the past while also making it possible to reanalyse already existing studies of seafaring that have been based on other methods.

**Grammar, spelling, etc.**

While the content is strong, the paper requires significant editorial work:

Figures:

Many figures are of poor resolution, making symbols and labels illegible (eg, Figures 2 and 3). Figures should be revised to ensure clarity and readability, with high-resolution exports suitable for publication. Response. All figures have been updated and issues highlighted by the reviewer has been corrected.

Also, the figure numbers found with the captions are inconsistent throughout the text, which was confusing for the reviewer. Response. Figure numbers have been corrected throughout the text.

Proofreading:

Numerous typographical errors (e.g., missing conjunctions, duplicated words) and overly long sentences detract from the paper’s clarity. Some of these have been marked in the text but there are others. Between this and the figure captions, this paper could benefit from a thorough revision and final proofing before publication. Response. Hopefully this is now addressed.

**Recommendation**

The study presents a promising contribution to seafaring archaeology but requires revisions before publication. Addressing the methodological concerns, situating the work within existing literature, and improving the presentation would significantly enhance its impact and reach.
